# Design, Synthesis and Activity of New *N*^1^-Alkyl Tryptophan Functionalized Dendrimeric Peptides against Glioblastoma

**DOI:** 10.3390/biom12081116

**Published:** 2022-08-13

**Authors:** Marta Sowińska, Monika Szeliga, Maja Morawiak, Barbara Zabłocka, Zofia Urbanczyk-Lipkowska

**Affiliations:** 1 Institute of Organic Chemistry PAS, 01-224 Warsaw, Poland; 2 Mossakowski Medical Research Institute PAS, 02-106 Warsaw, Poland

**Keywords:** dendrimers, glioblastoma, viability, colony-formation assay, reactive oxygen species

## Abstract

Background: Due to resistance to conventional therapy, a blood–brain barrier that results in poor drug delivery, and a high potential for metastasis, glioblastoma (GBM) presents a great medical challenge. Since the repertoire of the possible therapies is very limited, novel therapeutic strategies require new drugs as well as new approaches. The multiple roles played by *L*-tryptophan (Trp) in tumorigenesis of GBM and the previously found antiproliferative properties of Trp-bearing dendrimers against this malignancy prompted us to design novel polyfunctional peptide-based dendrimers covalently attached to *N*^1^-alkyl tryptophan (Trp) residues. Their antiproliferative properties against GBM and normal human astrocytes (NHA) and their antioxidant potential were tested. Methods: Two groups of amphiphilic peptide dendrimers terminated with *N*^1^-butyl and *N*^1^-aminopentane tryptophan were designed. The influence of dendrimers on viability of NHA and human GBM cell lines, displaying different genetic backgrounds and tumorigenic potentials, was determined by the MTT test. The influence of compounds on the clonogenic potential of GBM cells was assessed by colony-formation assay. Dendrimers were tested for radical scavenging potency as well as redox capability (DPPH, ABTS, and FRAP models). Results: Several peptide dendrimers functionalized with *N*^1^-alkyl-tryptophan at 5 µM concentration exhibited high selectivity towards GBM cells retaining 85–95% viable NHA cells while killing cancer cells. In both the MTT and colony-formation assays, compounds **21** (functionalized with *N*^1^-butyl-Trp and (+)8 charged) and **25** (functionalized with *N*^1^-aminopentane-Trp and (+)12 charged) showed the most promise for their development into anticancer drugs. According to ABTS, DPPH, and FRAP antioxidant tests, dendrimers functionalized with *N*^1^-alkylated Trp expressed higher ROS-scavenging capacity (ABTS and DPPH) than those with unsubstituted Trp. Conclusions: Peptide dendrimers functionalized with *N*^1^-alkyl-tryptophan showed varying toxicity to NHA, while all were toxic to GBM cells. Based on their activity towards inhibition of GBM viability and relatively mild effect on NHA cells the most advantageous were derivatives **21** and **25** with the respective di-dodecyl and dodecyl residue located at the C-terminus. As expected, peptide dendrimers functionalized with *N*^1^-alkyl-tryptophan expressed higher scavenging potency against ROS than dendrimers with unsubstituted tryptophan.

## 1. Introduction

Among various types of cancer experienced by humans, glioblastoma (GBM) is one of the most aggressive and most common malignancies of the central nervous system (CNS) [1,2]. Despite significant progress that has been made recently in the development of therapeutic strategies in cancer therapy, GBM demonstrates particular treatment challenges involving resistance to conventional therapy, localization in the brain that results in suppression of drug delivery by blood–brain barrier, high potential for metastasis, intratumoral heterogeneity at the molecular and cellular level, etc. [3,4,5]. In view of the significant tumor heterogeneity and diverse resistance mechanisms, not only novel therapeutic approaches but also an enhanced knowledge of molecular mechanisms related to disease genesis, progression, and growth is necessary [6,7,8,9].

Among many compounds that have emerged as promising novel chemotherapeutics are dendrimers [10] and branched, polyfunctional nanosized molecules [11,12,13]. Due to their particular structure they are proposed as vehicles in drug delivery as various nanocarriers [14,15,16] or as more complex structures combining therapeutic and diagnostic capability (theranostics) [17,18,19,20]. Recently, design of dendrimeric nanomolecules either with intrinsic anticancer activity alone or associated with the presence of chelated metal cations have emerged [21,22,23,24,25,26,27,28].

We have previously demonstrated that the second generation poly-lysine dendrimers terminated with 2-chlorobenzyloxycarbonyl (2-Cl-Z) residue inhibit the proliferation of GBM cell lines without expressing significant toxicity against non-tumoral CNS cells (neurons and glia) [29]. Cytotoxic effect was enhanced when dendrimers contained indole residue (side chain of tryptophan) at the C-terminal position. On the other hand, peptide dendrimers functionalized with *p*-aminobenzoic acid (PABA) expressed activity against the human melanoma cell line, also playing a protective role in neurons [30]. Moreover, dendrimers rich in *L*-tryptophan (Trp) on one side and containing PABA residues on the other side of the so-called non-symmetric bola structure effectively reduced cell proliferation and long-term colony formation propensity in GBM and neuroblastoma cell lines [31].

Trp is an aromatic amino acid that is often found in hydrophobic tails of peptides or membrane proteins spanning lipid bilayers [32]. Due to its hydrophobic character and ability to be involved in hydrogen bonding, pi–cation, and other non-covalent interactions it is often located at water/lipid bilayer interface, where it plays a role of an anchor and protein structure stabilizer [33,34].

Another recently discovered aspect of Trp biochemistry is the involvement of tryptophan catabolic products in the silencing response of the immune system. In particular, the oxidative enzyme indoleamine 2,3-dioxygenase (IDO) is capable of depleting tryptophan in tumor cells and the local microenvironment in favor of kynurenine. Trp disappearance and kynurenine accumulation cooperate to mediate immunosuppression in tumors and tumor-draining lymph nodes [35,36]. Thus, search for the selective IDO inhibitors became a new target in basic research and clinical GBM immunotherapy [37]. Among low-molecular-weight inhibitors of IDO, 1-methyl-*L*-tryptophan (1MT) was found to selectively restore T-cell proliferation, overcoming cells resistance to therapy [38,39]. Furthermore, Sun et al., reported significant cytotoxic properties for simple *N*^1^-alkyl-modified Trp derivatives [40]. We have shown previously, that accumulation of Trp residues in peptide dendrimers of so-called bola structure resulted in significant antioxidant and radical scavenging properties along with variable anticancer activity [31].

Therefore, we hypothesized that further chemical modification of Trp-rich dendrimers towards more hydrophobic structures may improve intracellular penetration along with anticancer activity. Since biological activity of dendrimers depends both on structure of the branched part and also on the number and spatial distribution of the active groups [10], fragments from the peptide dendrimers previously successfully exploited as anti-GBM agents (left side of the bola structure), have been used as scaffolds [31]. This new modification involved introduction at the *N*^1^-position of Trp, of either alkyl or alkyl-amino residue. While an alkyl chain increases molecular hydrophobicity, addition of alkyl-amino groups enhances their cationic character.

The two new series of dendrimers were evaluated for in vitro cell toxicity against primary human astrocytes and three GBM cell lines varying with respect to tumorigenic potential and molecular alterations (LN229, T98G, and U87MG) [41]. Studies were extended to testing dendrimers for their ability to influence colony formation process. These compounds were also tested for molecular antioxidant capacity by applying ferric reducing antioxidant power (FRAP), and radical-scavenging ability in 2,2-diphenyl-1-picrylhydrazyl (DPPH) and 2,2′-azino-bis(3-ethylbenzothiazoline-6-sulphonic acid) diammonium salt (ABTS) laboratory tests.

## 2. Materials and Methods

### 2.1. General Procedures

All solvents and reagents were of analytical grade and were used without further purification. All solvents were obtained from Sigma-Aldrich (Steinheim, Germany). Mass spectra were recorded with a Mariner ESI time-of-flight mass spectrometer (PerSeptive Biosystems, Foster City, CA, USA) for the samples prepared in MeOH. The ^1^H-NMR and ^13^C-NMR spectra were recorded using a Bruker Advance spectrometer (Karlsruhe, Germany) at 500/125 or 400/100 MHz, respectively, using deuterated solvents and TMS as an internal standard. Chemical shifts are reported as δ values in parts per million, and coupling constants are given in hertz. The optical rotations ([α]**_D_^25^**) were measured with JASCO J-1020 digital polarimeter (Ishikawa-machi, Hachioji, Tokyo, Japan). Melting points were recorded on a Köfler hot-stage apparatus (Wagner & Munz, München, Germany) and are uncorrected. Thin-layer chromatography (TLC) was performed on aluminum sheets with silica gel 60 F254 from Merck (Darmstadt, Germany). Column chromatography (CC) was carried out using silica gel (230–400 mesh) from Merck or Sephadex LH20 (Biosciences, Upsala, Sweden). The TLC spots were visualized by treatment with 1% alcoholic solution of ninhydrin and heating.

### 2.2. Synthesis of N^1^-Susbstituted Tryptophanes

**Scheme 1:**
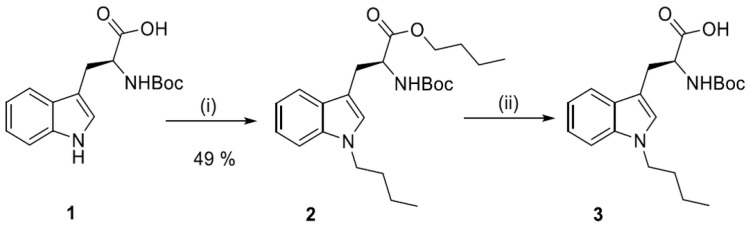
Synthesis of *N*^1^-Boc-1-butylotryptophan (**3**). (i) *n*-BuBr, NaOH, DMSO, 60 °C, 6 h; (ii) 1M NaOH, DMSO, r.t., 30 min.

The basic substrate i.e., *N*^1^-Boc-1-butylotryptophan (**3**), was obtained initially by the two-step procedure reported by Sun et al. [40]—i.e., one pot esterification and *N*^1^-alkylation of Boc-Trp-OH (**1**) with 3 equivalents of *n*-butyl bromide in the presence of NaOH (2 equation) in DMSO, followed by basic hydrolysis of the ester **2** (Scheme 1).

**Scheme 2:**
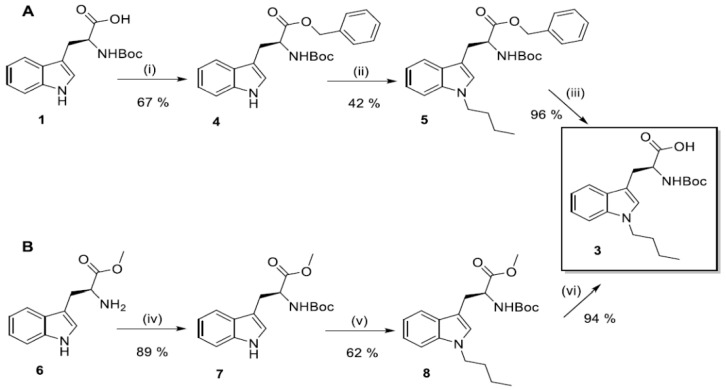
Synthesis of *N*^1^-Boc-1-butylotryptophan (**3**). (**A**)—(i) BnBr, Cs_2_CO_3_, DMF, rt., 6 h.; (ii) *n*-BuBr, NaH, DMF, 0 °C, 1.5 h.; (iii) H_2_/10% Pd-C, MeOH, 3.5 h.; (**B**)—(iv) (Boc)_2_O, NaOH, H_2_O/dioksan, r.t., 24 h; (v) *n*-BuBr, NaH, DMF, 0 °C, 2.5 h; (vi) (1) 1M NaOH, MeOH, 50 °C, 1.5 h; (2) 1M HCl.

Since the first reaction step was inefficient, (ester **2** was obtained in only 16% yield), product **3** was obtained according to a common procedure of indole *N*^1^-alkylation, either starting from substrate **1** or **6** (Scheme 2A or Scheme 2B).

**Scheme 3:**
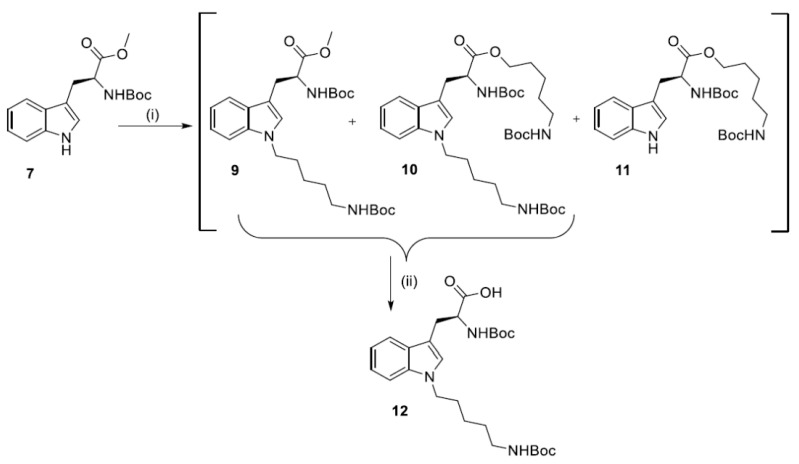
Synthesis of *N*^1^-Boc-1-[5-(Boc-amino)pentyl]tryptophan (**12**). (i) MeSO_2_O(CH2)_5_NHBoc, NaH, DMF, r.t., 24 h; (ii) (1) 1M NaOH, MeOH, 50 °C, 2 h; (2) 1M HCl.

Introduction of alkyl chain in *N*^1^-position of Trp increases the lipophilic character of the resulting dendrimers. An increase of a number of positive charges was another modification that was also considered. Therefore, the *N*^1^-position of Trp was substituted with 5-(Boc-amino)pentyl residue (Scheme 3).

Synthesis of *N*^1^-Boc-1-[5-(Boc-amino)pentyl]tryptophan (**12**) was performed via *N*^1^-alkylation of Boc-Trp-OMe (**7**) with 5-(Boc-amino)pentyl mesylate and NaH at room temperature for 24 h (Scheme 3). The crude product obtained by extraction was immediately hydrolyzed in basic conditions. The pure product *N*^1^-Boc-1-[5-(Boc-amino)pentylo]tryptophan (**12**) was obtained in 35.8% yield by silica column chromatography. It was a highly hygroscopic white powder with 105.9–108.4 °C melting point.

### 2.3. Synthesis of Dendrimers Functionalized with N^1^-Alkyl Tryptophanes

**Scheme 4:**
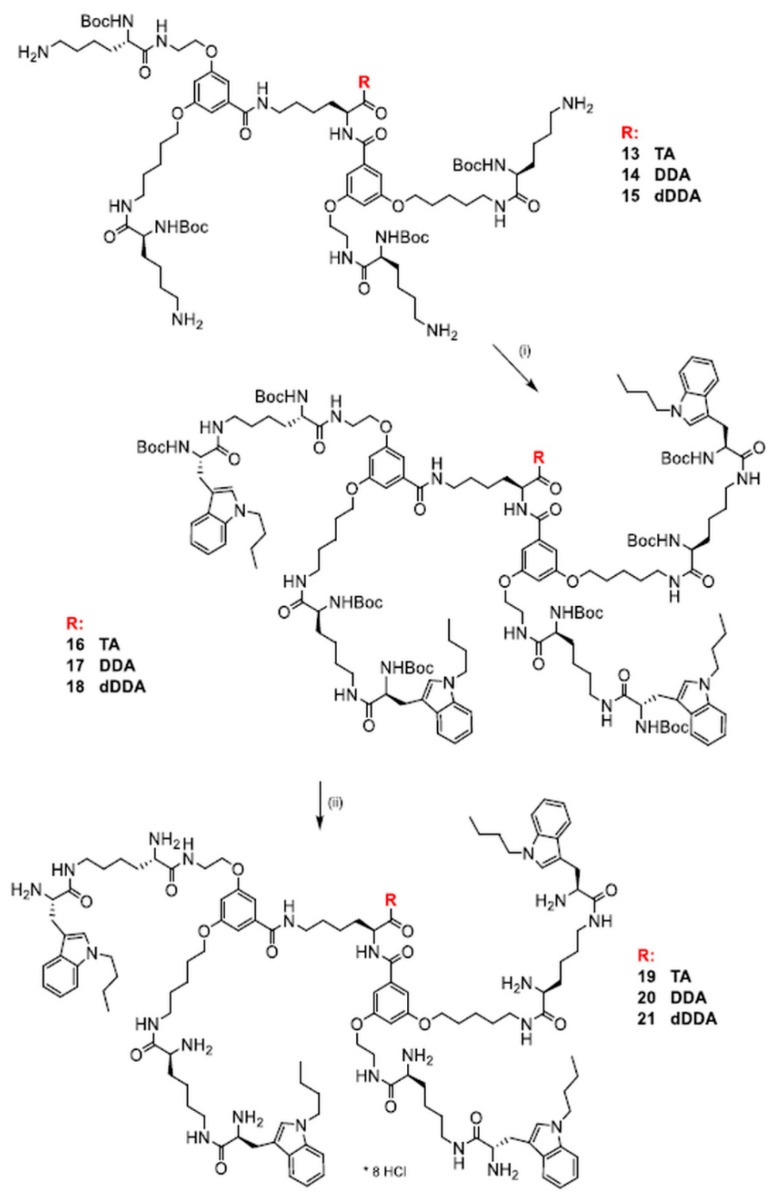
Synthesis of dendrimers with terminal *N*^1^-butyltryptophan residue (**16**–**18** and **19–21**), where TA, DDA, and dDDA denote tryptamine, dodecylamine, and di-dodecylamine, respectively; (i) **3**, EDC/HOBt, Et_3_N, DMF, 48–96 h; (ii) sat. HCl/EtOAc.

Synthesis of the final dendrimers functionalized with *N*^1^-butyltryptophan was performed by coupling the previously described [42], intermediate dendrimers **13**, 1**4**, and **15** with *N*^1^-Boc-1-butylotryptophan (**3**) using EDC/HOBt in DMF with an additional amount of Et_3_N (3.6 equation) (Scheme 4). Size-exclusion chromatography with Sephadex LH-20 column and MeOH as eluent, followed by silica-gel chromatography using CHCl_3_/MeOH (100:1→15:1 gradient) provided dendrimers **16**–**18** with yields in the range 35.8–71.4% (Table 1). *N*^1^-Boc-deprotection was accomplished by dissolution of the dendrimer in the minimal amount of MeOH and treatment with saturated solution of HCl in EtOAc until disappearance of the substrate (Scheme 4). Solvents were evaporated and the remaining solid was treated several times with MeOH and evaporated to remove an excess of HCl. Finally, size-exclusion chromatography was applied with a Sephadex LH-20 filled column and MeOH as eluent. Dendrimers were kept in desiccator over P_2_O_5_. Deprotected dendrimers **19**–**21** are hygroscopic octa-hydrochlorides obtained in 89.8–97.6% yield range (Table 1).

Analytical data, i.e., ESI MS spectra with mass assignments, ^1^H and ^13^C NMR spectra along with chemical shifts assignment, molar ellipticity, and melting points for the final dendrimers are available in Supplementary Materials.

**Scheme 5:**
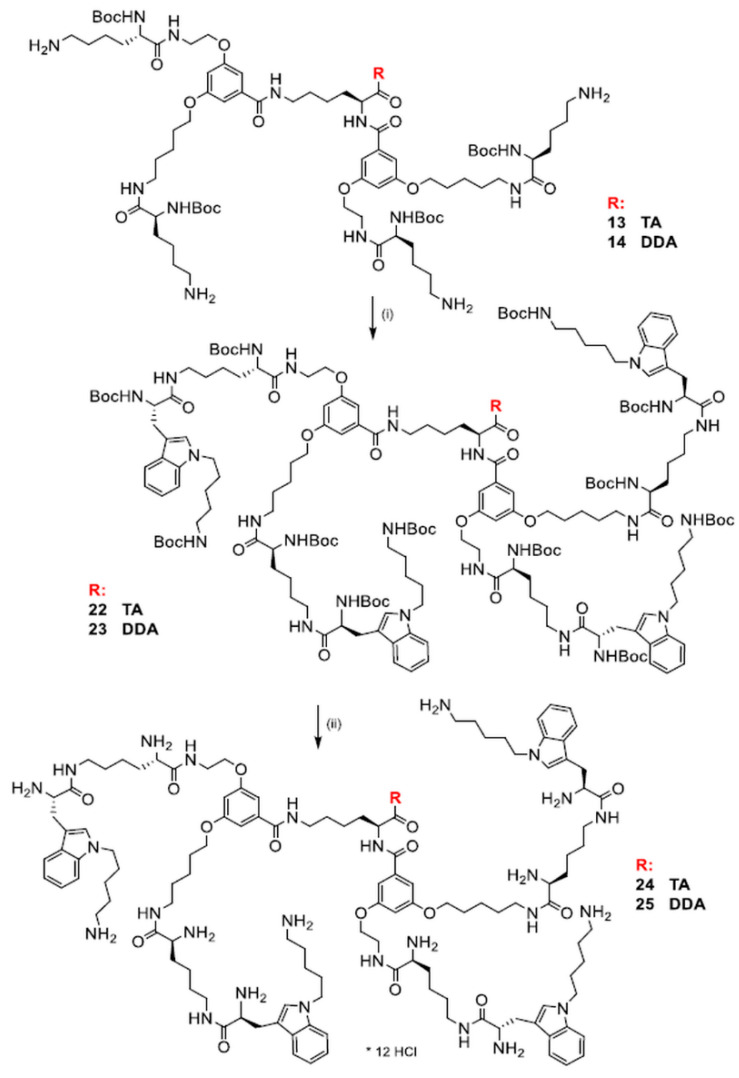
Synthesis of dendrimers terminated with 1-(5-aminopentyl)tryptophan (**22**, **23,** and **24**, **25**, where TA, and DDA denote tryptamine and dodecylamine, respectively; (i) **12**, EDC/HOBt, Et_3_N, DMF, 96 h; (ii) sat. HCl/EtOAc.

Synthesis of dendrimers terminated with (5-aminopentyl)tryptophan residues was performed in the same conditions yielding the respective dendrimers **24** and **25** in total yields not exceeding 22% (Scheme 5).

Boc-protected dendrimers were initially purified by size-exclusion chromatography on Sephadex LH-20 filled column using MeOH as eluent. Finally, silica-gel chromatography using CHCl_3_/MeOH with (100:1→15:1 gradient) was applied providing Boc-protected dendrimers **22**–**23** with the respective yields 68.1 and 78.9% (Table 1). *N*^1^-Boc- deprotection was performed by dissolution of the dendrimer in the minimal amount of MeOH and treatment with saturated solution of HCl in EtOAc, followed by the same purification route as in the case of dendrimers **19**–**21**, i.e., multiple evaporation of MeOH to remove HCl, followed by size-exclusion chromatography and drying over P_2_O_5_ (Scheme 5). Deprotected dendrimers **24**–**25** are hygroscopic dodeca-hydrochlorides obtained in the respective 89.5 and 97.8% yields (Table 1). The final dendrimers were characterized by electrospray mass spectrometry (ESIMS) and ^1^H and ^13^C NMR spectrometry. ^1^H and ^13^C NMR spectra showed no extra peaks suggesting at least 95% purity of the final dendrimers.

### 2.4. Human GBM Cell Lines and Human Astrocytes Culture

T98G human GBM cell line purchased from ATCC (American Type Culture Collection, Manassas, VA, USA) was maintained in Minimum Essential Medium Eagle (MEM) (Sigma-Aldrich, St. Louis, MO, USA) supplemented with 10% fetal bovine serum (FBS) (Gibco, Thermo Fisher Scientific, Grand Island, NY, USA), 50 U/mL penicillin (Gibco), 50 μg/mL streptomycin (Gibco), and 1% non-essential amino acids (Gibco). U87MG cell line purchased from Sigma-Aldrich was maintained in Eagle’s Minimum Essential Medium (EMEM) (ATCC) supplemented with 50 U/mL penicillin, 50 μg/mL streptomycin, and 15% FBS (Gibco). LN229 cell line (kindly provided by Rafał Krętowski, PhD, Department of Pharmaceutical Biochemistry, Medical University of Białystok, Poland) was maintained in Dulbecco’s Modified Eagle Medium (DMEM) (Gibco) and supplemented with glucose (final concentration 4.5 g/L), 10% FBS, 50 U/mL penicillin, and 50 μg/mL streptomycin (Gibco). The cells were routinely tested for mycoplasma contamination using Mycoplasma Detection Kit-Quick Test (Biotool, Stratech Scientific Limited, Cambridge, UK). The used passage numbers were below 25. Normal human astrocytes (NHA) were purchased from ScienCell Research Laboratories (Carlsbad, CA, USA) and cultured in the astrocyte medium according to the manufacturer’s instruction. The cells were incubated at 37 °C, 5% CO_2,_ and 95% humidity.

### 2.5. Cell Survival

Cell survival was determined by 3-(4,5-dimethylthiazol-2-yl)-2,5-diphenyl tetrazolium bromide (MTT) conversion into formazan. Briefly, the cells were seeded into 24-well plates at a density of 4 × 10^4^ cells/well and incubated overnight at 37 °C. After this time, the cells were treated with increasing concentrations (1, 5, 20 µM) of dendrimers (**19**, **20**, **21**, **24**, and **25**) or dimethyl sulfoxide (DMSO) (Sigma-Aldrich) (the final concentration of DMSO did not exceed 0.1% *v*/*v*). After a 1 h incubation at 37 °C, the dendrimer-containing medium was replaced with the fresh one and the cells were cultured for the next 48 h. Subsequently, the cells were washed with phosphate-buffered saline (PBS) (Sigma-Aldrich) and incubated in the culture medium with MTT (Sigma-Aldrich) solution at the final concentration of 0.5 mg/mL for 2 h at 37 °C. Then, the medium was replaced with 100% DMSO and absorbance was read at 570 nm using Elisa Bio-Rad Microplate Reader (Bio-Rad, Hercules, CA, USA). Each compound in each concentration was tested in triplicate in a single experiment and five independent experiments were performed.

### 2.6. Colony-Forming Assay

LN229, U87MG, or T98G cells were seeded in six-well plates (100 cells/well) and allow to attach for 24 h. Next, cells were treated with increasing concentrations (1, 5, 20 µM) of dendrimers (**19**, **20**, **21**, **24,** or **25**) or DMSO (Sigma-Aldrich) for 1 h. Subsequently, the medium was replaced with a fresh one and cells were cultured for 14 days. Next, cell culture plates containing colonies were gently washed with PBS (Sigma-Aldrich) and fixed with 4% formaldehyde for 10 min. Colonies were stained with 0.5% crystal violet solution in 25% methanol for 10 min and the grossly visible colonies were counted. Three independent experiments were performed.

### 2.7. Statistical Analysis

Results are reported as means and standard deviations. The statistical analysis was performed using GraphPad Prism 7 (GraphPad Software, La Jolla, CA, USA). The statistical significance was determined by one-way analysis of variance (one-way ANOVA). * *p* < 0.05; ** *p* < 0.01; *** *p* < 0.005 was considered as statistically significant.

### 2.8. Antioxidant Assays

#### 2.8.1. Chemicals

2,4,6-tris(2-pyridyl)-s-triazine (TPTZ), iron(III) chloride hexahydrate, acetic acid (≥99.0%), sodium acetate, 6-hydroxy-2,5,7,8-tetramethylchromane-2-carboxylic acid (Trolox^®^), HCl, 2,2′-azino-bis(3-ethylbenzothiazoline-6-sulphonic acid) diammonium salt (ABTS), potassium persulfate, and 2,2-diphenyl-1-picrylhydrazyl radical (DPPH), were purchased from Sigma-Aldrich (Steinheim, Germany). All solvents (methanol and water) and reagents that are used in this study were HPLC or of analytical grade.

#### 2.8.2. Ferric Reducing Antioxidant Power (FRAP)

The FRAP assay was performed according to previously described method [43] with some modifications [44]. Fresh FRAP solution was prepared each time. The stock solution included 2.40 mL of acetate buffer (300 mM, pH 3.6), 0.24 mL 10 mM TPTZ solution in 40 mM HCl, and 0.24 mL of 20 mM FeCl_3_ × 6H_2_O in water. The mixture was protected from light and kept at 37 °C before using a 240 μL sample. Trolox or solvent (blank sample) standard solution was added to FRAP stock solution. The absorbance was measured at 593 nm after 15 min. Final concentration of methanolic solutions of the studied dendrimers were in the range 0–24 μM and Trolox^®^ solutions from 0 to 50 μM. All determinations were carried out in triplicate and the data are presented as Trolox-equivalent activity (TEAC).

#### 2.8.3. ABTS Assay

ABTS assay was based on a previously published method [45] with some modifications [46], using Trolox^®^, a water-soluble analogue of vitamin E, as a standard. ABTS^•+^ radical cation was produced by dissolution 10 mg solid ABTS in a mixture of water and 2.4 mM potassium persulfate aqueous solution (final concentration of ABTS 2 mM). The stock solution was kept in the dark at room temperature for 12–16 h. The ABTS^•+^ solution was then diluted with distilled water to obtain an absorbance of 1.05 ± 0.05 at 734 nm. To the 240 μL sample, solvent or Trolox standard solution, 2.88 mL of ABTS^•+^ solution was added, and absorbance was measured after 10 min. Results of the ability of dendrimers to scavenge cation radical ABTS are presented as Trolox-equivalent antioxidant capacity (TEAC) and as IC_50_ parameter. All analyses were performed in triplicate and the results were expressed as the mean value ± standard deviation.

#### 2.8.4. DPPH Assay

Experiments were performed according to [47], with small modifications [46]. Primarily, 2 × 10^−3^ mol/L of DPPH radical reagent was prepared in methanol. This solution was kept in the dark at room temperature for 2 h. The DPPH^•^ solution was then diluted with methanol to obtain an absorbance of 0.95 ± 0.05 at 517 nm. 240 μL of the solvent, sample or Trolox standard solutions were mixed with 2.88 mL of DPPH radical solution and after 15 min, the absorbance were measured. Results were expressed as Trolox-equivalent antioxidant capacity (TEAC) values (μM). In the concentration range investigated, 50% of radical scavenge (IC_50_) was not achieved. All experiments were repeated in triplicate and the results were shown as the mean value ± standard deviation.

## 3. Results

### 3.1. Molecular Design

**Figure 1:**
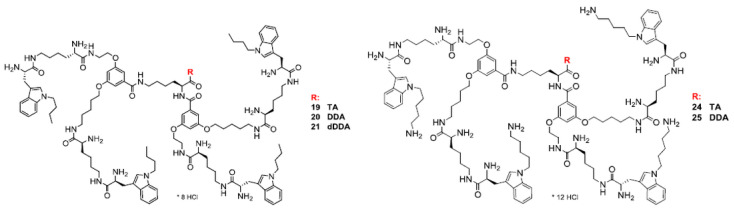
Chemical structure of the designed dendrimers.

Previously, we have studied several groups of dendrimers bearing 1 to 5 indole residues that, depending on the dendrimer geometry, expressed antimicrobial [42] or anticancer properties [31]. For some of them significant antioxidant capacity was detected along with ability to protect glutamate stressed primary rat cerebellar neurons [30]. We anticipated that dendrimeric structure will potentiate effect of a single residue, making observation of biological effect more visible. The present design involved synthesis of two groups of dendrimers with hydrophobic interior and indole *N*^1^-atom substituted with *N*^1^-alkyl (**19**–**21)** or *N*^1^-alkyl-amino residue (**24**–**25**) (Figure 1). The C-terminal position was substituted by different hydrophobic residues, including an aromatic indole ring from tryptamine or lipophilic dodecylamine (DDA) or di-dodecylamine (DDDA). Since compounds were prepared as poly-hydrochlorides, various protonation levels were achieved: (+)8 charged compounds **19**–**21** and (+)12 charged compounds **24**–**25**. The final dendrimers were characterized by electrospray mass spectrometry (ESIMS) and ^1^H and ^13^C NMR spectrometry. ^1^H and ^13^C NMR spectra showed no extra peaks suggesting at least 95% purity of the final dendrimers (Supplementary Materials). It was of interest to see if such molecular architecture could modulate selectivity of dendrimers towards GBM *versus* the non-tumorigenic cells or their molecular antioxidant activity. 

### 3.2. Susceptibility of Human GBM Cells and Primary Human Astrocytes to Treatment with Dendrimers

**Figure 2:**
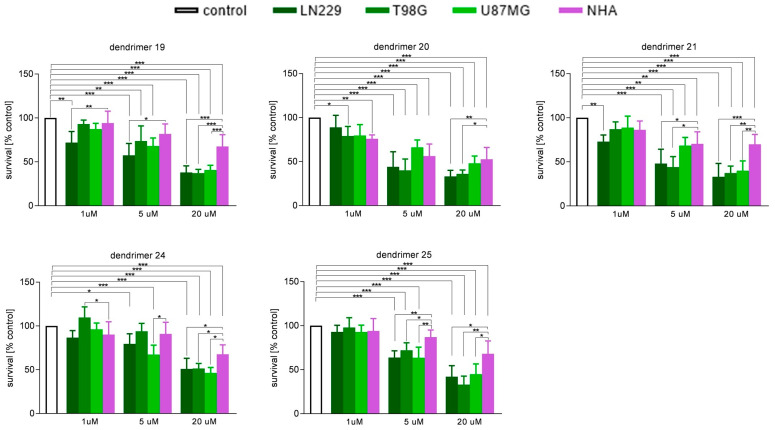
Influence of dendrimers on viability of human glioblastoma LN229, T98G, and U87MG cells and primary astrocytes. Cells were treated with increasing concentrations (1, 5, and 20 µM) of dendrimer **19**, **20**, **21**, **24**, and **25** for 1 h. Subsequently, the dendrimer-containing medium was replaced with the fresh medium. After the next 48 h, cells survival was assessed by MTT assay. Data are presented as mean ± SD (*n* = 5) expressed as percentage of control (DMSO-treated cells). * *p* < 0.05; ** *p* < 0.01; *** *p* < 0.005 (one-way ANOVA).

**Figure 3:**
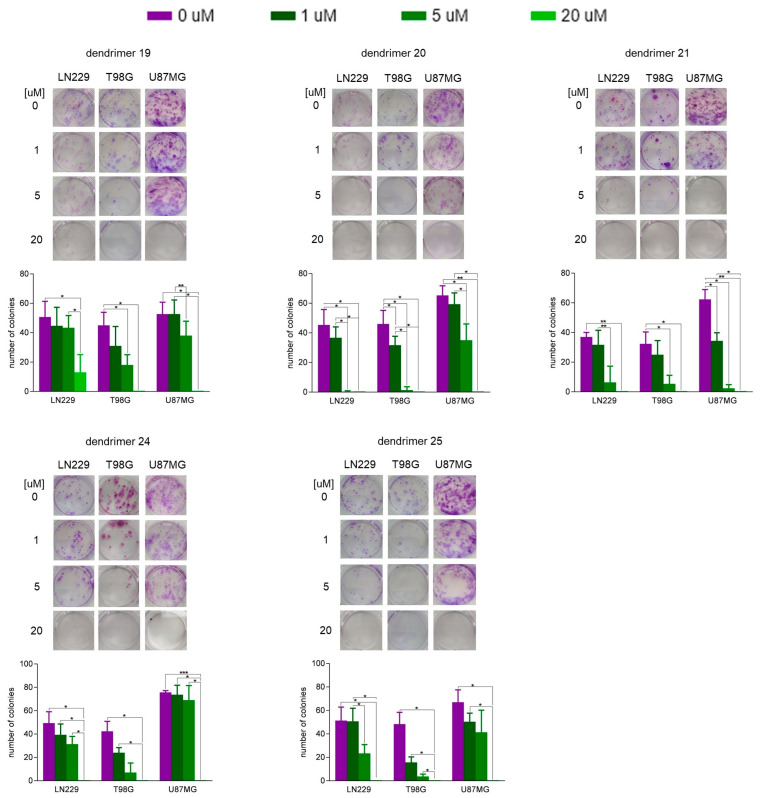
Influence of dendrimers on clonogenic potential of human glioblastoma LN229, T98G, and U87MG cells. Cells were treated with increasing concentrations (1, 5, and 20 µM) of dendrimer **19**, **20**, **21**, **24**, and **25** for 1 h. Subsequently, the dendrimer-containing medium was replaced with the fresh medium. After the next 14 days, colonies were stained and counted. Data are presented as mean ± SD (*n* = 3). * *p* < 0.05; ** *p* < 0.01; *** *p* < 0.005 (one-way ANOVA).

To assess the optimal dendrimer concentration providing anticipated cytotoxic activity in the malignant cells with minimal negative impact on the normal nervous system cells, viability of human normal astrocytes (NHA) and GBM cells was tested in vitro. Cells were cultured for 1 h in the presence of dendrimers belonging to two structural groups (**19**–**21**) and (**24** and **25**) in various concentrations. As shown in Figure 2, there was no dependence of protonation of dendrimers on viability of NHA assessed 48 h after the challenge. There was even an inverse relationship: (+)12 charged are less damaging to NHA than (+)8 if we compare compounds in 5 µM concentration. On the other hand, all molecules tested similarly reduced viability of GBM cells (Figure 2). In the colony-forming test (14 days after 1 h exposure to dendrimers), the most efficient in inhibition of cell proliferation were compounds **20**, **21**, **24** and **25**, as shown on Figure 3. While only dendrimers **21** and **25** were simultaneously safe for NHA (Figure 2) and harmful for GBM cells (Figure 3), it can be assumed that this behavior was, rather, related to the hydrophobicity of the compounds. The tested compounds were versatile in their ability to inhibit the division of three different types of GBM.

### 3.3. Antioxidant Properties: Radical Scavenging Potency (DPPH and ABTS) and Redox Potential (FRAP) of Dendrimers

Redox potential and radical scavenging potency of the new groups of peptide dendrimers functionalized with *N*^1^-substituted Trps were evaluated. Generally, two types of tests can be used to study antioxidant capacity of any individual compound or natural isolates. The first, and also the most common is the ferric reducing antioxidant power (FRAP) test that is based on the electron transfer (ET) mechanism and reflects the compound’s ability to reduce Fe(III) cation to the lower oxidation state Fe(II). The second group of assays involves interactions of compounds with free radicals: neutral radical scavenging assay shows capability of a compound to interact with stable neutral radicals prepared from 1,1-diphenyl-2-picrylhydrazyl (DPPH), whereas the second test shows scavenging power against cationic radicals prepared from 2,2′-azino-bis(3-ethylbenzothiazoline-6-sulphonic acid) (ABTS). Tests of the latter group use different models of antioxidative mechanism. While DPPH assay shows the capacity of antioxidant to transfer electron, ABTS assay determines cationic free radical scavenging activity involving both electron and hydrogen transfer mechanisms. In all tests, the reference compound is Trolox, a known radical scavenger.

**Figure 4:**
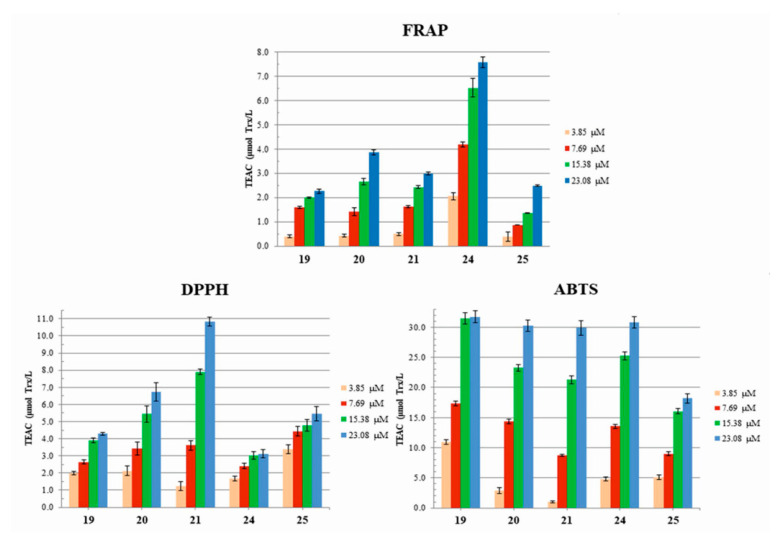
Antioxidant potential expressed as Trolox-equivalent activity (TEAC) of the selected **19**–**21** and **24**, **25** dendrimers at four concentrations (3.85, 7.69, 15.38, and 23.08 µM) measured as ferric reducing antioxidant power (FRAP), neutral radicals scavenging ability (DPPH), or cationic radicals scavenging ability (ABTS) in relation to the known concentrations of Trolox.

The antioxidant potential in terms of FRAP model for the studied compounds: +(8) charged **19**–**21** (*N*^1^-butyl-Trp terminated arms and tryptamine (TA), dodecylamine (DDA) or *di*-dodecylamine (DDDA) residue located at C-terminus)) and +(12) charged **24** and **25** (four *N*^1^-aminopentyl-Trp terminated arms and TA or DDA residue located at C-terminus) is shown in Figure 4. From the collection of dendrimers only compound **24** expressed a significantly higher TEAC value than the other compounds.

The results of DPPH and ABTS tests for the studied group of dendrimers at four concentrations (3.78, 7.69, 15.38, and 23.08 µM) are shown in Figure 4. Considering absolute TEAC values, individual dendrimers showed different level of scavenging capacity in both tests, being generally better scavengers of radical cations than single-electron-bearing neutral radicals. In the ABTS model a two-fold increase in dedrimer concentration yielded, as expected, ca. double TEAC values, except for **19** and **25,** where, for the highest concentration (23.08 µM), increase was marginal, suggesting that compounds approached Trolox activity.

## 4. Discussion

The amino-acid-containing dendrimers presented here are branched version of amphiphilic, natural peptides (AMP) known for several decades as primary defense molecules of the innate immune system. In addition to their numerous functions (antimicrobial, antifungal, antiprotozoal, immunomodulatory, etc.), anticancer properties of AMPs are of great importance [48,49,50,51,52].

Design of the present Trp-rich peptide dendrimers with *N*^1^-atom substituted with *N*^1^-alkyl (**19**–**21**) or *N*^1^-alkylamino residue (**24**–**25**) (Figure 2) was prompted by multiple functions played by Trp, e.g., structural stabilization of the spanning membrane peptides and proteins [32,33,34], antioxidant properties [53,54], and involvement of its catabolic products in development of immunosuppression in cancer disease [35,36,37]. According to the current knowledge, natural peptides express variability of anticancer mechanisms, including membrane disintegration and induction of apoptosis by reactive-oxygen-species accumulation. Our own studies on Trp-rich amphiphilic dendrimeric peptides of the bola structure revealed moderate activity against GBM cells [31].

The goal of this study was to evaluate the influence of five newly designed peptide dendrimers on human GBM cells. Bearing in mind a significant heterogeneity of GBM, we used three cell lines varying with respect to molecular background and tumorigenic potential [41]. All tested dendrimers reduced viability and clonogenic potential of GBM cells to different extents. The differences in sensitivity to each of the compounds observed between particular cell lines most likely result from the aforementioned biodiversity of cell lines used in this study. Indeed, several groups have documented a differential response of those three cell lines to various therapeutic approaches [55,56,57,58]. Here, compared to T98G and LN229 cells, U87MG cells turned out to be less susceptible to dendrimer-mediated toxicity, which was particularly pronounced in colony-forming assay. In comparison to U87MG, T98G cells display high expression of multidrug resistance protein 1 (MRP1), which is one of the representative members of the ATP-binding cassette superfamily of transporters that is involved in resistance to chemotherapeutic agents in cancer patients [59]. However, as showed by Kang et al., 2005 alpha-tocopheryl succinate (TOS), a vitamin E analogue, decreased intracellular GSH concentration and blocked MRP1 function in T98G, so, co-treatment of TOS and anticancer drug etoposide sensitized T98G cells to the drug more efficiently then U87MG cells [59]. In our analysis, T98G and LN229 cells were sensitive to the tested dendrimers, so we may assume that their action may be related to GSH and inhibition of MRP1. Basically, variation in cell responses to the tested compounds may result from different genetic modifications of the cells. As shown in 34 randomly chosen human glioma cell lines, the status of *TP53*, *p16*, *p14ARF*, and *PTEN* tumor-suppressor genes could participate in biological pathways that are functioning separately/independently in glioma cells and did not correlate with tumorigenicity or any clinical parameters [41]. Clearly, further studies are required to elucidate the reasons of this varied sensitivity of different GBM cells to the dendrimers as well as their mechanisms of action. The problem of delivering drugs to the brain is important because the blood–brain barrier is impermeable to most compounds. However, nanoparticles (<1000 nm), including dendrimers, may pass through or bypass the BBB depending on how the compound is administered, as detailed in the Hersh et al., 2022 review [60].

It is worth emphasizing, that a potential anti-glioma drug should display cytotoxic activity in tumor cells without affecting normal cells. In this study, dendrimers **21** and **25** turned out to simultaneously be safe for NHA and harmful for GBM cells. Moreover, as compound **24** was the most toxic to all cells in the MTT and colony formation tests, and at the same time displayed the greatest iron reduction ability in the FRAP test, we can speculate that the selective toxicity of **21** and **25** dendrimers to GBM is probably not due to this property.

Another important issue which has to be taken into consideration is the time of treatment with potential drugs and mode of action. The effect of 1 h treatment of cells with new dendrimers on their viability, as assessed 48 h later, or on clonogenicity observed after 14 days was shown by us. In the paper by Khoei et al., 2016, U87MG cells cultured in spheroids were treated with the well-known antioxidant resveratrol (20 µM) alone or in combination with iododeoxyuridine (IUdR 1 µM) for 67 h without any effect on cell viability or clonogenicity observed 10 day later. However, combined treatment of resveratrol and IUdR sensitized cells to subsequent irradiation with 60Co (2 Gy) [61]. Resveratrol (trans-3,4,5′-trihydroxystilbene) inhibits HIF1-a protein expression in the hypoxia condition in cancer cells [62]. It can therefore be assumed that the new dendrimers exerted toxic effects on GBM during short-term treatment in a different mechanism than that of resveratrol during the long-term and poorly effective treatment of U87MG cells.

Metabolically controlled accumulation of ROS, typical for cancer cells, induces oxidative stress and may lead to such pro-tumorigenic processes as phospholipid oxidation, DNA instability, and production of disadvantageous metal cations at a higher oxidation state. Furthermore, recent evidence has accumulated that ROS also have a role as signaling molecules in regulation cancer-cell proliferation, metastasis, angiogenesis, etc., [63,64] or inducing cancer-cell apoptosis [3,4,5,53,54,65]. It can be speculated that introduction of exogenous chemotherapeutics with antioxidant activity would affect GBM cells in a positive or negative way. Since the unsubstituted indole ring present in Trp and indolamine has antioxidant properties [66,67] an important issue is contribution of *N*^1^-alkyl residues to stabilization of the generated Trp cation radicals, (presumably by (1-*n*) hydrogen atom transfer mechanism (1-*n* HAT)) [68], in terms of magnitude and biological response of GBM cells. Dendrimers were tested for antioxidant capacity focusing on three models—ferric reducing antioxidant power (FRAP), interactions with free radicals: neutral radical scavenging assay (DPPH), and scavenging power against cationic radicals (ABTS).

Interestingly, in the DPPH model, TEAC values increased with an increase of basicity of the C-terminal nitrogen atom in the dDDA > DDA > TA fashion, whereas in ABTS model this trend was reversed (Figure 4). Taking into account structural differences between these two groups of dendrimers, the possible primary mechanism in the DPPH conditions involves hydrogen and electron transfer from the above electron-rich center and to less extent from numerous indole rings. For the same reasons, the highest reductive capabilities (FRAP) were expressed by compound **24**. All compounds were very effective in scavenging the ABTS cation radical. The magnitude of the dendrimer’s redox potential was visible by comparison with the known data for clinically used alpha-tocopherol and resveratrol. As shown by Gülçin at al., 2010, radical scavenging potency of alpha-tocopherol at ca 4.4 µM concentration (relevant to our 3.85 µM), is slightly lower and ca 5.5 higher than potency of Trolox in the respective DPPH and ABTS models [69]. Resveratrol was much more potent than Trolox in both tests. Since none of our compounds expressed that level of activity one might assume that their involvement in enhancing ROS production was mild. Instead, they might slightly reduce the amount of ROS generated by GBM cells. Previously, an important role of ROS generation in TOS-induced, caspase-independent apoptosis of cancer cells was shown [70]. However, human lung cancer A549 and H460 cells were much more sensitive to TOS-induced apoptosis than the T98G and U87MG cells indicating that ROS generation is not the primary destruction mechanism for GBM. Moreover, exploratory metabolomics study detected elevated serum levels of a panel of molecules with antioxidant properties as well as oxidative-stress-generated compounds [71]. This study points out a latent biomarker for future glioblastoma consisting of γ-tocopherol, α-tocopherol, erythritol, erythronic acid, myo-inositol, cystine, 2-keto-L-gluconic acid, hypoxanthine, and xanthine, all involved in antioxidant metabolism. Unfortunately, the mechanisms of association between the serum metabolite pattern and future GBM development are not known.

As found by Su et al., 2020, the number and distribution of cationic centers is another important aspect during development of an active secondary structure of a linear anticancer peptides [52]. In the present case, enhanced cationicity of compounds **24** and **25** did not correlate with an increased activity. This might originate from the effect of back-folding, well known in dendrimer chemistry, i.e., H-bonding interactions of the *N*^1^-aminopentyl residue with hydrogen-bond acceptors located inside of the dendrimer molecule that might change molecular availability for interactions with cell membranes [72].

As shown in Table 1 (Materials and Methods), hydrophobicity of dendrimers is mostly defined by lipophilicity of the substituent located at C-terminal position. For the *N*^1^-Boc protected dendrimers, the retention factor (Rf) visualized on the TLC plate with the use of (CHCl3/MeOH (8:1)) solvent system increased in the order: tryptamine < dodecylamine < di-dodecylamine, i.e., 0.46, 0.55, and 0.60, for the respective compounds **19**, **20**, and **21**. Substitution of tryptophan *N*^1^-atom by 5-aminopentane residue resulted in small decrease of Rf, as shown in the first group, i.e., 0.44 vs. 0.46 (compound **10** vs. **24**) and 0.55 vs. 0.52 (compound **20** vs. **24**).

**Figure 5:**
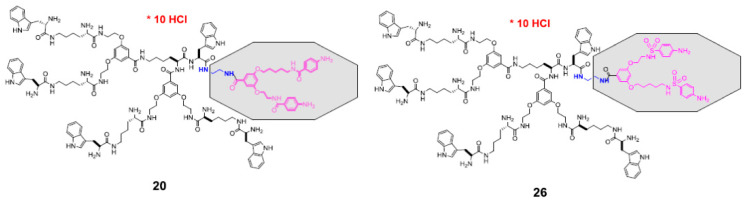
Structures of the selected compounds from [31]: originally compounds **20** and **26**, showing left side of the bola structure that represents Trp-unsubstituted version of the present compounds **19**—**21** and **24**, **25**. Right side is functionalized with two *p*-aminobenzoic acid (PABA, **20**) or *p*-aminobenzenosulfonic acid (PAS, **26**) residues.

In the present group of compounds, the radical-stabilization effect gained due to (1-*n* HAT) mechanism should increase TEAC values in both DPPH and ABTS models, when compared to the two previously studied compounds with similar structures shown in Figure 5 [31]. Indeed, at 15.38 µM dendrimer concentration, averaged TEAC values reached ca. 7.9 in DPPH model and ca. 30 µM Trolox/L in ABTS model, in comparison with TEAC’s for the bola dendrimers that reached values 5.5 and 25 µM Trolox/L, for the DPPH and ABTS models, respectively. This factor might be important if electrons annihilating radicals produced by GBM are transferred from tryptophanes.

Summing up, the results of three tests indicated that functionalization of dendrimer scaffolds with either *N*^1^-butyl-Trp or *N*^1^-aminopentyl-Trp increases their antioxidant potential, when compared to dendrimers with unsubstituted Trp [31]. Moreover, compounds described here are versatile in their ability to inhibit the division of three different types of GBM and have a mild influence on normal astrocytes. It is worth mentioning that these compounds display higher anti-GBM activity compared to aforementioned dendrimers with unsubstituted Trp, which were tested in our previous study [31].

It should be noted that the MTT and colony-forming assays differ from each other not only in terms of duration, but they also examine different phenomena. While the MTT test evaluates cell metabolism, the colony-forming assay examines cell clonogenicity. Since compounds **21** and **25** turned out to be toxic towards GBM cells in both assays, and relatively not harmful to NHA, it is tempting to speculate that they are the most promising anti-GBM derivatives. However, the dendrimer’s toxicity is not correlated with their antioxidant potential. Therefore, the next step is to investigate whether these compounds enter the cells or interact with the cell membranes as can be suggested by the high lipophilicity and positive charge of the molecules tested. Both factors contribute to the mechanism of anticancer activity expressed by some natural antimicrobial peptides [49].

## 5. Conclusions

We tested the potential of a rationally designed novel series of dendrimeric peptides to reduce survival and hold back the clonogenic potential of three GBM cell lines. The results showed that exposure of GBM cells to dendrimers for one hour has a short and long-term effect—significantly high cytotoxicity when measured after 48 h and arrest of colony formation detected after 14 days.

These study highlighted the major aspects of molecular design. Dendrimers with hydrophobic interior and lipophilic substituents: DDA and DDDA located at C-terminus exhibit moderate toxicity in GBM cells compared to NHA. Although *N*^1^-alkylation of Trp yielded derivatives with enhanced radical scavenging activity, their antioxidant potency was lower than that of the clinically used α-tocopherol and resveratrol.

## Figures and Tables

**Table 1:** Physicochemical data for dendrimers **16**–**25**.

*N*^1^-Protected Dendrimers					Final Dendrimers			
No	Yield (%)	m.p. (°C)	(α)__D_^25^ (c 1, MeOH)	R_f_ (CHCl_3_/MeOH 8:1)	No	Yield. (%)	m.p. (°C)	(α)_D_^25^ (c 1, MeOH)
**16**	56.6	120–122	−6.5	0.46	**19**	97.6	182–184.5	+18.0
**17**	35.8	102–104.5	−5.4	0.55	**20**	89.8	174–177	+12.3
**18**	71.4	94–97	−7.1	0.60	**21**	95.8	175–178	+8.9
**22**	78.9	117–119.5	−6.5	0.44	**24**	97.8	189–192	+6.3
**23**	68.1	99–102	−5.7	0.52	**25**	89.5	184–187	+6.6

## Data Availability

Not applicable.

## Supplementary Materials

The following supporting information can be downloaded at: https://www.mdpi.com/article/10.3390/biom12081116/s1, S1: Synthesis and analytical data.

## Abbreviations

CNS	central nervous system
GBM	glioblastoma
ROS	reactive oxygen species
NHA	normal human astrocytes
