# Supplementary material for: Design, Synthesis and Activity of New N1-Alkyl Tryptophan Functionalized Dendrimeric Peptides against Glioblastoma
Source: Biomolecules. 2022 Aug 13;12(8):1116. doi: 10.3390/biom12081116 (PMC9406037; doi:10.3390/biom12081116)
Supplement: Supplementary file 1 [file biomolecules-12-01116-s001.zip › biomolecules-1863580-SI.pdf]

Article

Design, Synthesis and Activity of New N^1-Alkyl Tryptophan Functionalized Dendrimeric Peptides against Glioblastoma

Marta Sowińska [1,†], Monika Szeliga [2,†,*], Maja Morawiak [1], Barbara Zabłocka [2] and Zofia Urbanczyk-Lipkowska [1,*]

[1] Institute of Organic Chemistry PAS, 01-224 Warsaw, Poland
[2] Mossakowski Medical Research Institute PAS, 02-106 Warsaw, Poland
* Correspondence: mszeliga@imdik.pan.pl (M.S.); zofia.lipkowska@icho.edu.pl (Z.U.-L.)
† These authors contributed equally to this work.

Supplementary Material S1

S1.1. Synthesis of N-alkyl derivatives of tryptophan, dendrimers and their analytical data

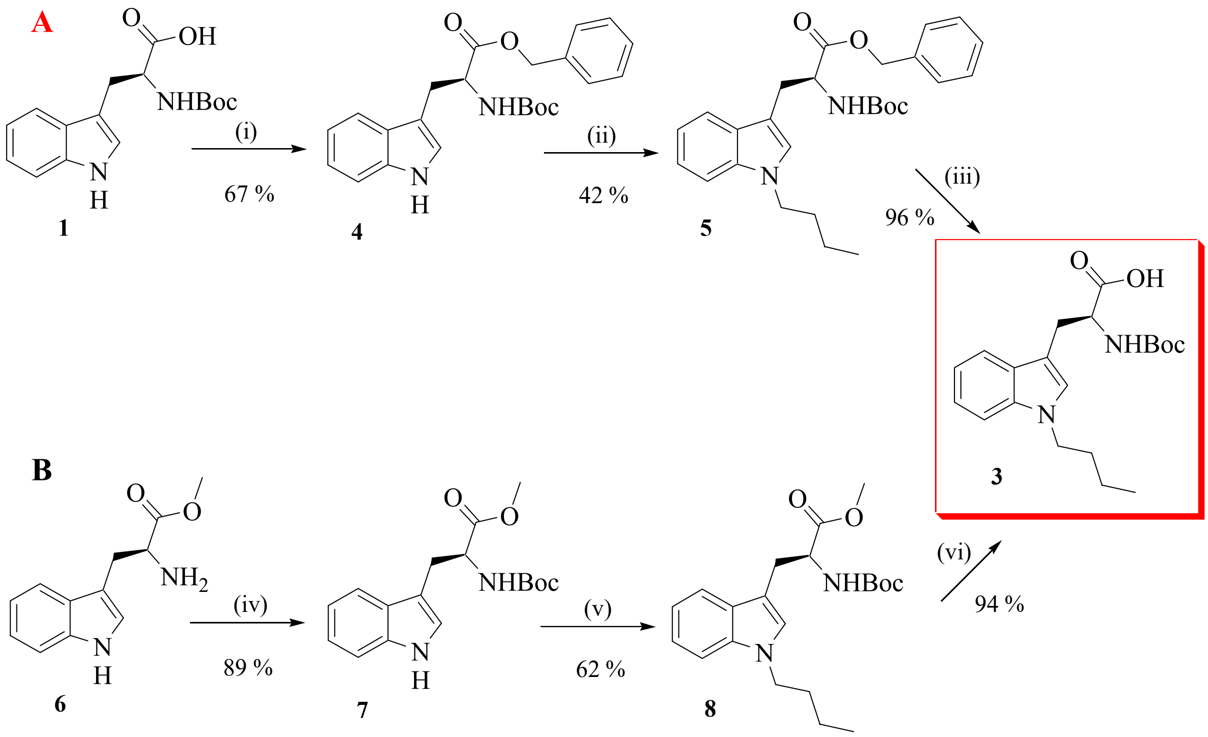

Scheme S1. Synthesis of N^1-Boc-1-butylotryptophan (**3**). A – (i) BnBr, Cs_2CO_3, DMF, rt., 6 h.; (ii) n-BuBr, NaH, DMF, 0 °C, 1.5 h.; (iii) H_2/10% Pd-C, MeOH, 3.5 h.; B – (iv) $(Boc)_2O$, NaOH, H_2O/dioksan, r.t., 24 h; (v) n-BuBr, NaH, DMF, 0 °C, 2.5 h; (vi) 1) 1M NaOH, MeOH, 50 °C, 1.5 h; 2) 1M HCl.

N-Boc-1-butylotryptofan (3)

Alcaline hydrolysis of 2.27 g (6.06 mmol, 1 eq.) of the ester **8** dissolved in 40 mL MeOH and addition of 12 mL (2 eq.) of 1 M NaOH was performed for 1.5 h. Product **3** was obtained in the form of colorless oil which after drying in dessicator over P_2O_5 solidified to a white powder; yield 94% (2.05 g).

$C_{20}H_{28}O_4N_2$, M = 360.45 (monoisotopic mass 360.2).

LRMS (ESI, EtOAc): 743.4 [2M + Na$^+$], 383.3 [M + Na$^+$] - *main signals*, 361.5 [M + H$^+$], 283.4 [M – Boc + Na$^+$], 719.7 [2M – H$^-$, *negative ions*] - *main signals*, 359.4 [M – H$^-$, *negative ions*].

$[\alpha]_D^{25}$ = +7.8 (c 1, MeOH), -6.8 (c 1, 1 M NaOH).

R_f = 0.42 ($CHCl_3$/MeOH 8:1).

m.p.: 120-122.3˚C.

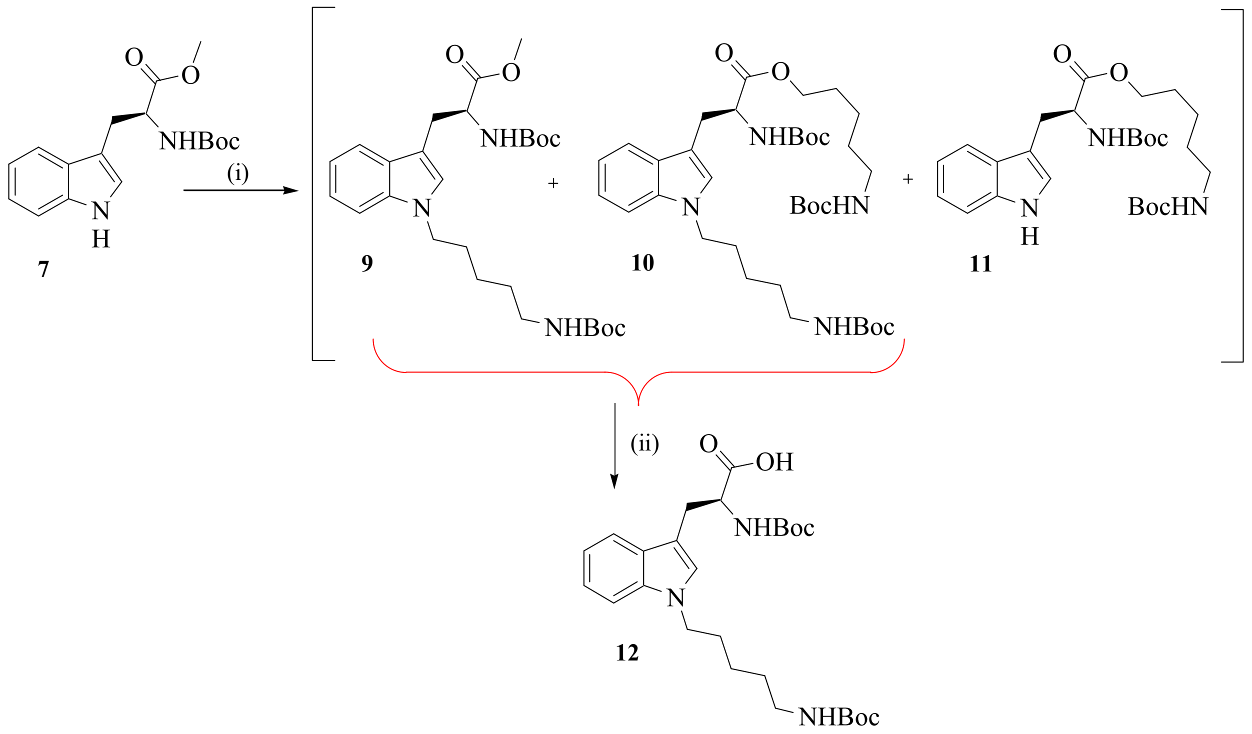

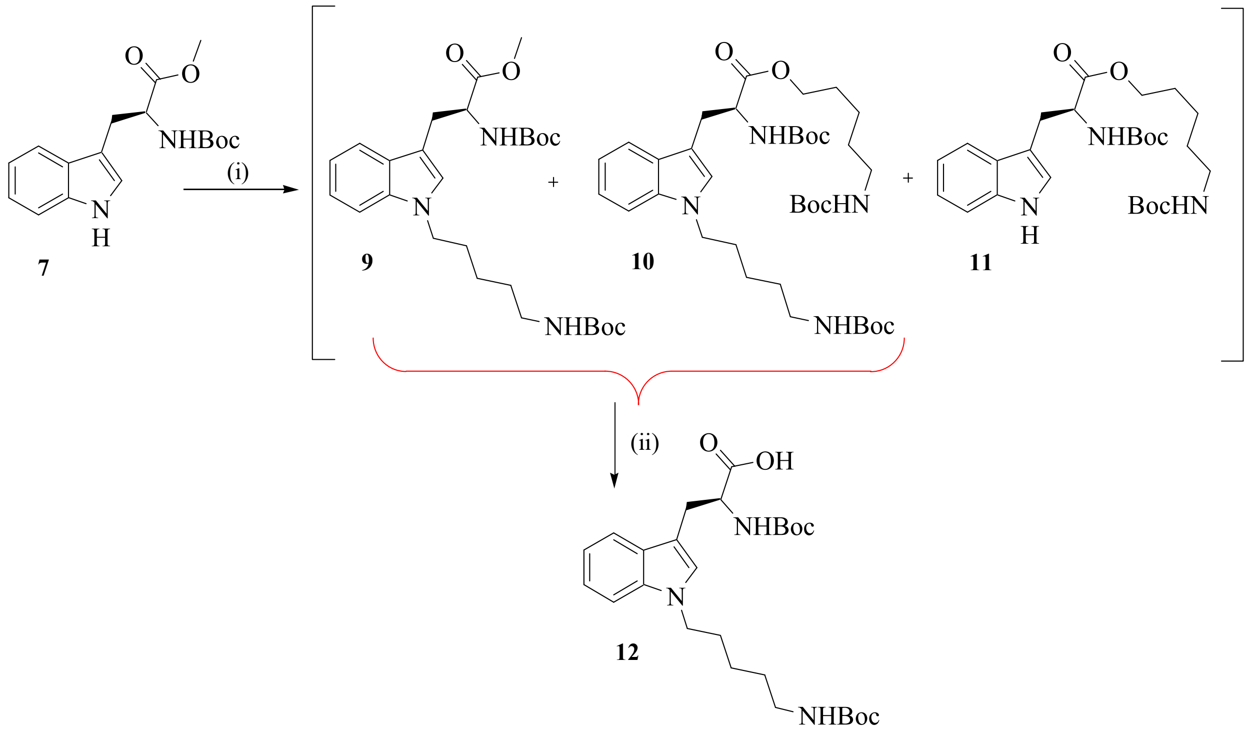

Scheme S2. Synthesis of *N*-Boc-1-[5-(Boc-amino)pentyl]tryptophan (**12**). (i) $MeSO_2O(CH2)_5NHBoc$, NaH, DMF, r.t., 24 h; (ii) 1) 1M NaOH, MeOH, 50 °C, 2 h; 2) 1M HCl.

b) *N*-Boc-1-[5-(Boc-amino)pentyl]tryptophan (12)

To a suspension of NaH (301.6 mg, 7.54 mmol, 1.2 eq., 60% suspension in mineral oil) in 10 mL DMF, cooled to 0 °C, solution of Boc-Trp-OMe (**7**, 2 g, 6.28 mmol, 1 eq.) in 15 mL DMF was added and stirred for 30 min. Then reaction mixture was warmed to r.t. and three portions of 5-(Boc-amino)pentyl mesylate (**8**, 3.53 g, 12.56 mmol, 2 eq.) in 10 mL DMF were added. Then temperature was raised to 50 °C and stirring was continued for 24 h. After this time reaction mixture was poured down to 40 mL of a cold water and extracted with EtOAc (3 × 30 mL). Organic phase was rinsed with H_2O (20 mL) and brine (20 mL) and dried over $MgSO_4$. Solvent evaporation yielded yellow oil, which was dissolved in 100 mL MeOH. To this solution 30 mL of 1 M water solution of NaOH was added and stirred for 2h in 50 °C. Reaction mixture was evaporated and 40 mL of H_2O was added. Mixture was acidified with 1 M HCl to pH = 2 and extracted with EtOAc (3 × 20 mL). Organic phase was dried over $MgSO_4$ and the solvent was evaporated. Crude product was purified with two steps of silica gel chromatography. First with $CHCl_3$/MeOH (gradient 100:1→15:1), and second with EtOAc/hexane (gradient 1:9→3:7), and finaly eluted with EtOAc. Pure product was obtained as a white powder that was dried over P_2O_5 (1.1 g, 35.8%) yield.

$C_{26}H_{39}O_6N_3$, M = 489.60 (monoisotopic mass 489.3).

LRMS (ESI, MeOH): 544.4 [M + MeOH + Na^+], 534.4 [M + $2Na^+$ - H^-]$^+$, 528.3 [M + K^+], 512.5 [M + Na^+] - *main signals*, 490.4 [M + H^+].

^{1}H NMR (500 MHz, MeOD): δ = 1.17-1.51 [br m, 22H, 2×$C(CH_3)_3$, CH_2-3,4 *5-AP*], 1.80 (m, 2H, CH_2-2 *5-AP*), 2.98 (br t, 2H, CH_2-5 *5-AP*), 3.10 (m, 1H, βCH_2), 3.27 (m, 1H, βCH_2), 4.11 (br t, 2H,CH_2-1 *5-AP*), 4.40 (m, 1H, αCH), 7.01 (br t, 1H, C^5-H), 7.04 (s, 1H, C^2-H), 7.12 (br t, 1H, C^6-H), 7.32 (d, J = 8.18 Hz, 1H, C^7-H), 7.56 (d, J = 7.83 Hz, 1H, C^4-H).

^{13}C NMR (500 MHz, MeOD): δ = 25.1 (C^3 *5-AP*), 28.4 (βC), 28.6, 28.8 [2×C($\boldsymbol{C}H_3)_3$], 30.6 (C^4 *5-AP*), 31.0 (C^2 *5-AP*), 41.2 (C^5 *5-AP*), 46.8 (C^1 *5-AP*), 56.0 (αC), 79.8, 80.5 [2×$\boldsymbol{C}(CH_3)_3$], 110.5 (C^7), 110.6 (C^3), 119.8 (C^5), 119.9 (C^4), 122.4 (C^6), 127.9 (C^2), 129.6 (C^{3a}), 137.7 (C^{7a}), 157.7, 158.5 [2×C=O (Boc)], 175.8 (CO_2H).

$[\alpha]_D^{25}$ = +1.3 (c 1, MeOH).

R_f = 0.47 ($CHCl_3$/MeOH 8:1).

m.p.: 105.9-108.4˚C.

S1.2. Analytical data for the new dendrimers

Dendrimer 19

Dendrimer 19 was obtained with 97.6% (0.41 g) yield from 0.5 g (0.16 mmol) of dendrimer **16**. as creamy powder.

$C_{128}H_{184}O_{15}N_{24}$×8HCl, M = 2590.67 (monoisotopic mass of non-protonated dendrimer - 2298.3).

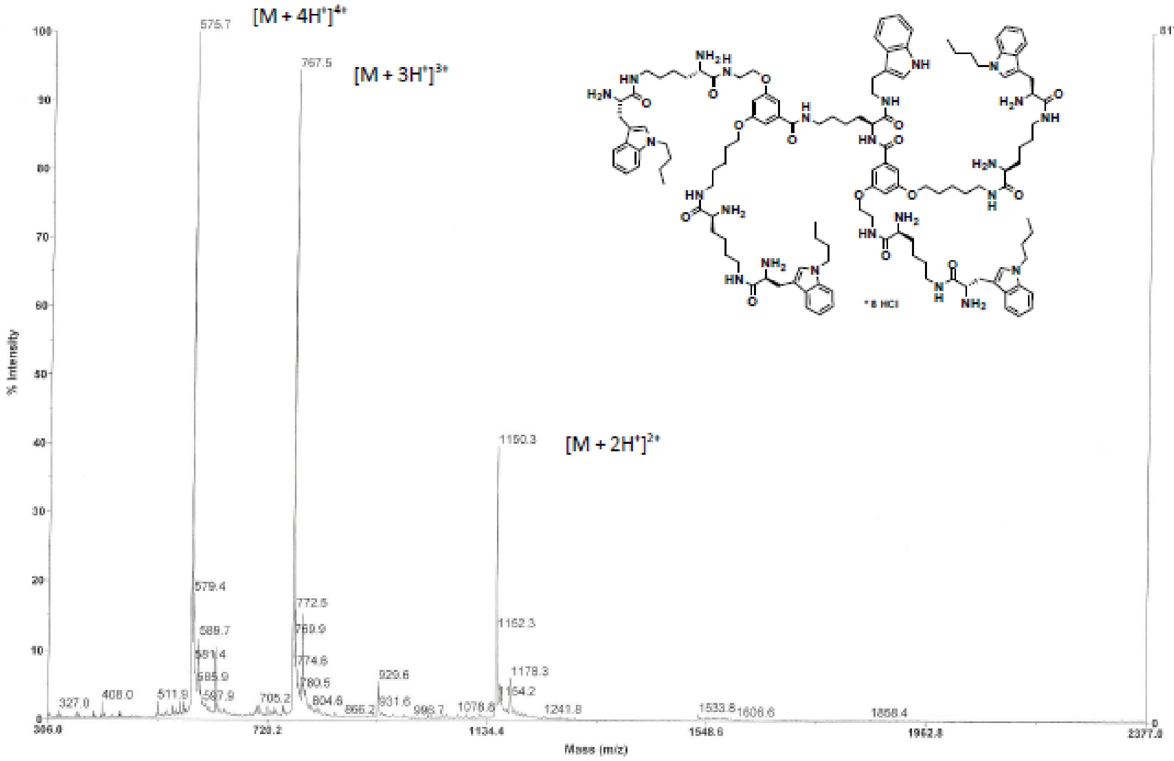

LRMS (ESI, MeOH): 1149.8 $[M + 2H^+]^{2+}$, 766.9 $[M + 3H^+]^{3+}$, 575.4 $[M + 4H^+]^{4+}$ - *main signals*.

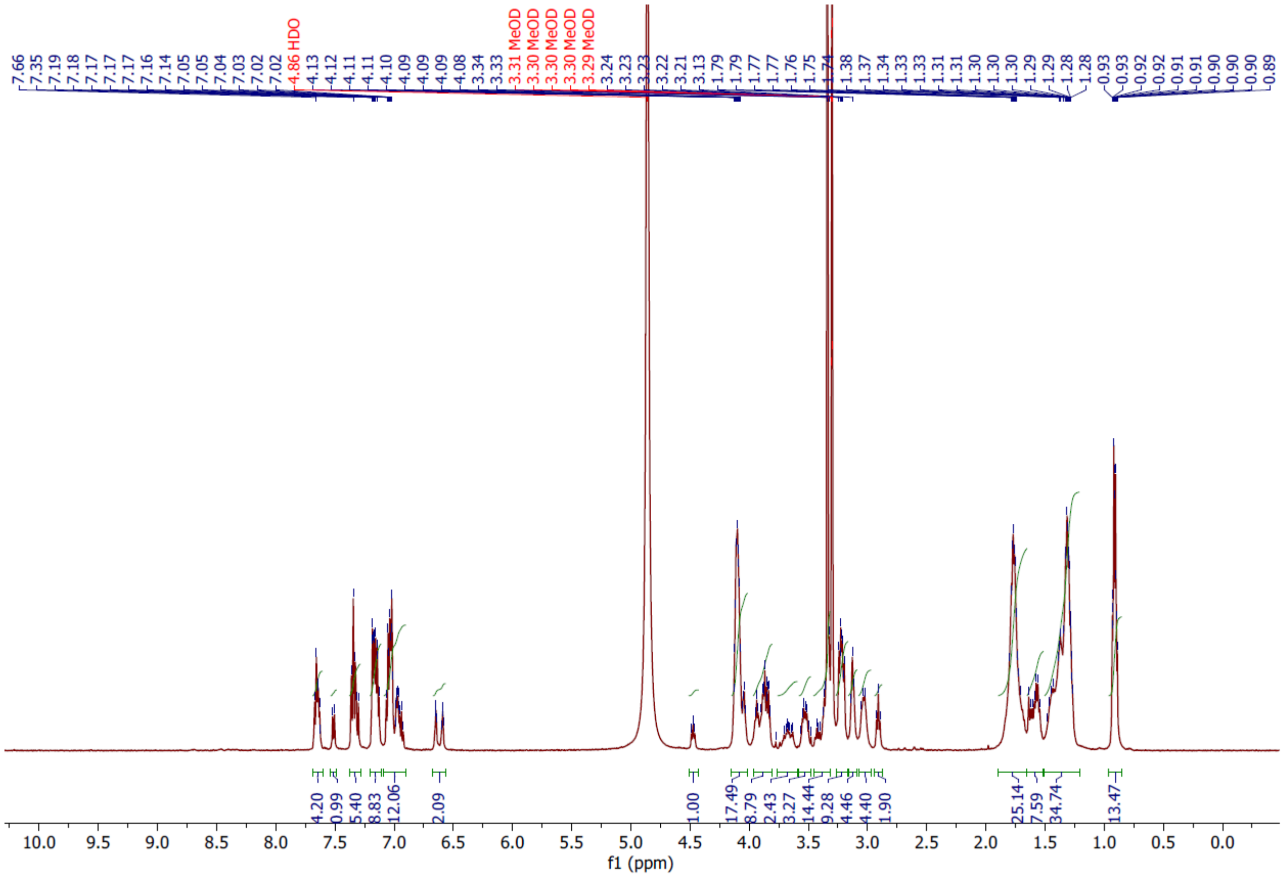

^{1}H NMR (500 MHz, MeOD): δ = 0.91 (m, 12H, 4×CH_3 *n-Bu*), 1.24-1.86 [br m, 58H, 5×β, γ, δCH_2 *L-Lys* and *core*, 2×O-CH_2-(C$\boldsymbol{H_2}$)$_3$-CH_2-NH, 4×CH_2-2,3 *n-Bu*], 2.91 (t, J = 7.25 Hz, 2H, CH_2-Ar *TA*), 3.03 [m, 4H, 2×O-(CH_2)$_4$-C$\boldsymbol{H_2}$-NH], 3.13 (m, 4H, 2×εCH_2 *L-Lys*), 3.19-3.40 (br m, 14H, 3×εCH_2 *L-Lys i core*, βCH_2 *B-Trp*), 3.42 (m, 1H, C$\boldsymbol{H_2}$-NH *TA*), 3.53 (m, 3H, O-CH_2-C$\boldsymbol{H_2}$-NH, C$\boldsymbol{H_2}$-NH *TA*), 3.67 (m, 2H, O-CH_2-C$\boldsymbol{H_2}$-NH), 3.81-3.98 [br m, 8H, 2×O-C$\boldsymbol{H_2}$-(CH_2)$_4$-NH, 4×αCH *L-Lys*], 4.02-4.15 (br m, 16H, 4×αCH *B-Trp*, 2×O-C$\boldsymbol{H_2}$-CH_2-NH, 4×CH_2-1 *n-Bu*), 4.48 (m, 1H, αCH *core*), 6.59, 6.65 (2m, 2H, C^4-H *Ph*), 6.91-7.08 (br m, 11H, $C^{2,6}$-H *Ph*, C^5-H *B-Trp*, $C^{2,5,6}$-H *TA*), 7.17 (m, 8H, $C^{2,6}$-H *B-Trp*), 7.34 (m, 5H, C^7-H *B-Trp*, C^7-H *TA*), 7.51 (d, J = 6.85 Hz, 1H, C^4-H *TA*), 7.65 (m, 4H, C^4-H *B-Trp*).

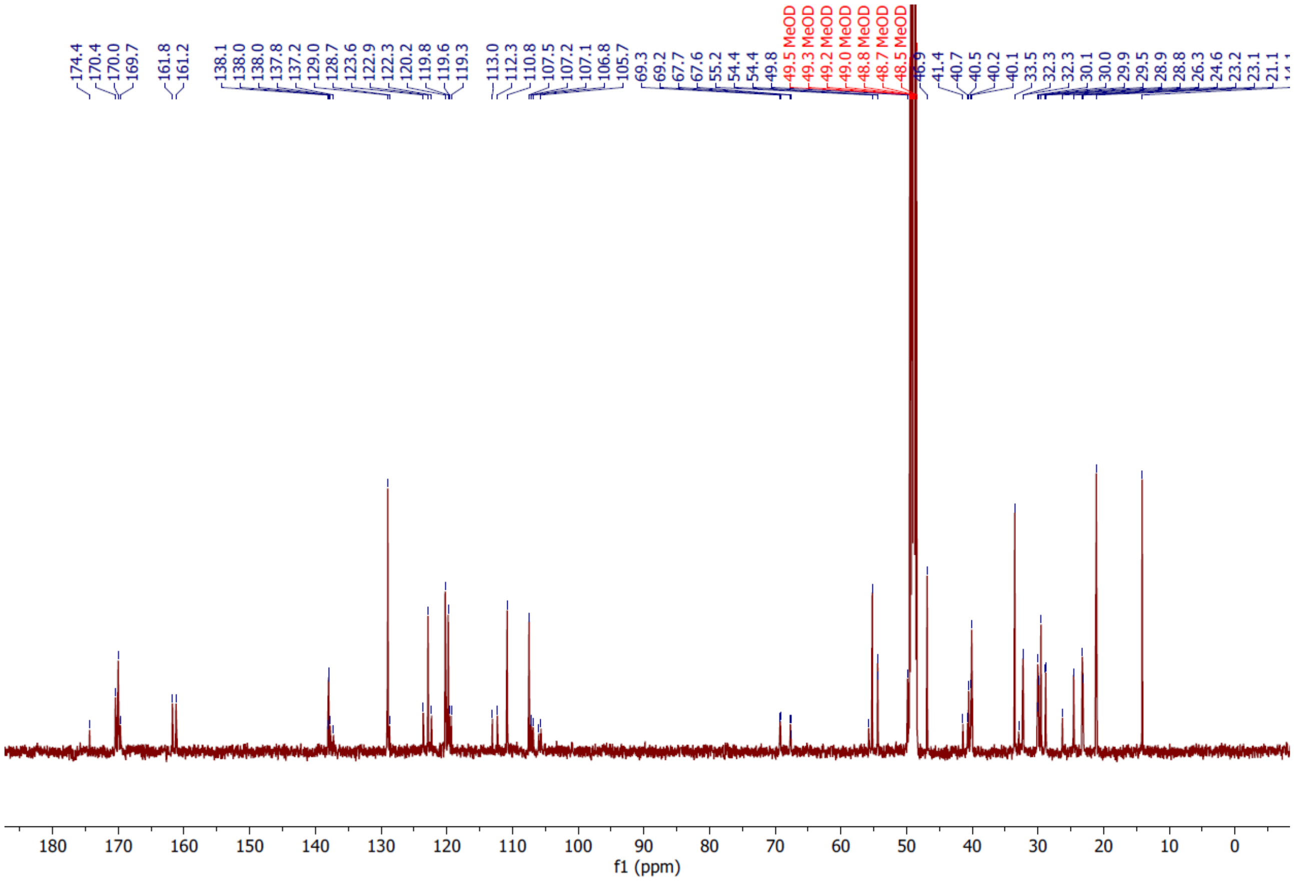

^{13}C NMR (125 MHz, MeOD): δ = 14.1 (C^4 *n-Bu*), 21.1 (C^3 *n-Bu*), 23.1, 23.2, 24.6 [γC, 2×O-$(CH_2)_2$-***C***H_2-$(CH_2)_2$-NH], 26.3 (***C***H_2-Ar *TA*), 28.8, 28.9 (βC *B-Trp*), 29.5, 29.9, 30.0, 30.1 [δC, 2×O-CH_2-***C***H_2-CH_2-***C***H_2-CH_2-NH], 32.3, 33.5 (βC, C^2 *n-Bu*), 40.1 (εC *L-Lys*), 40.2 (2×O-CH_2-***C***H_2-NH), 40.6 [2×O-$(CH_2)_4$-***C***H_2-NH], 40.7 (εC *core*), 41.4 (CH_2-NH *TA*), 46.9 (C^1 *n-Bu*), 54.4 (4×αC *L-Lys*), 55.2 (4×αC *B-Trp*), 55.8 (αC *core*), 67.6, 67.7 (2×O-***C***H_2-CH_2-NH), 69.2 [2×O-***C***H_2-$(CH_2)_4$-NH], 105.7, 106.0 (C^4 *Ph*), 106.8, 107.1, 107.5 ($C^{2,6}$ *Ph*), 110.8 (C^7 *B-Trp*), 112.3 (C^7 *TA*), 113.0 (C^3 *TA*), 119.3 (C^4 *TA*), 119.6 (C^4 *B-Trp*), 119.8 (C^5 *TA*), 120.2 (C^5 *B-Trp*), 122.3 (C^6 *TA*), 122.9 (C^6 *B-Trp*), 123.6 (C^2 *TA*), 128.7 (C^{3a} *B-Trp*), 129.0 (C^2 *B-Trp*), 138.0 (C^{7a} *B-Trp*), 138.1 (C^{7a} *TA*), 161.2, 161.8 ($C^{3,5}$ *Ph*), 170.0, 170.1, 170.5 (CONH).

$[\alpha]_D^{25}$ = +18.0 (c 1, MeOH).

m.p.: 182-184.5˚C.

Dendrimer 20

Dendrimer **20** was obtained with 89.8% (0.15 g) yield from 0.2 g (0.064 mmol) of dendrimer **17** in the form of creamy powder.

$C_{130}H_{199}O_{15}N_{23}$×8HCl, M = 2615.80 (monoisotopic mass of non-protonated dendrimer - 2324.1).

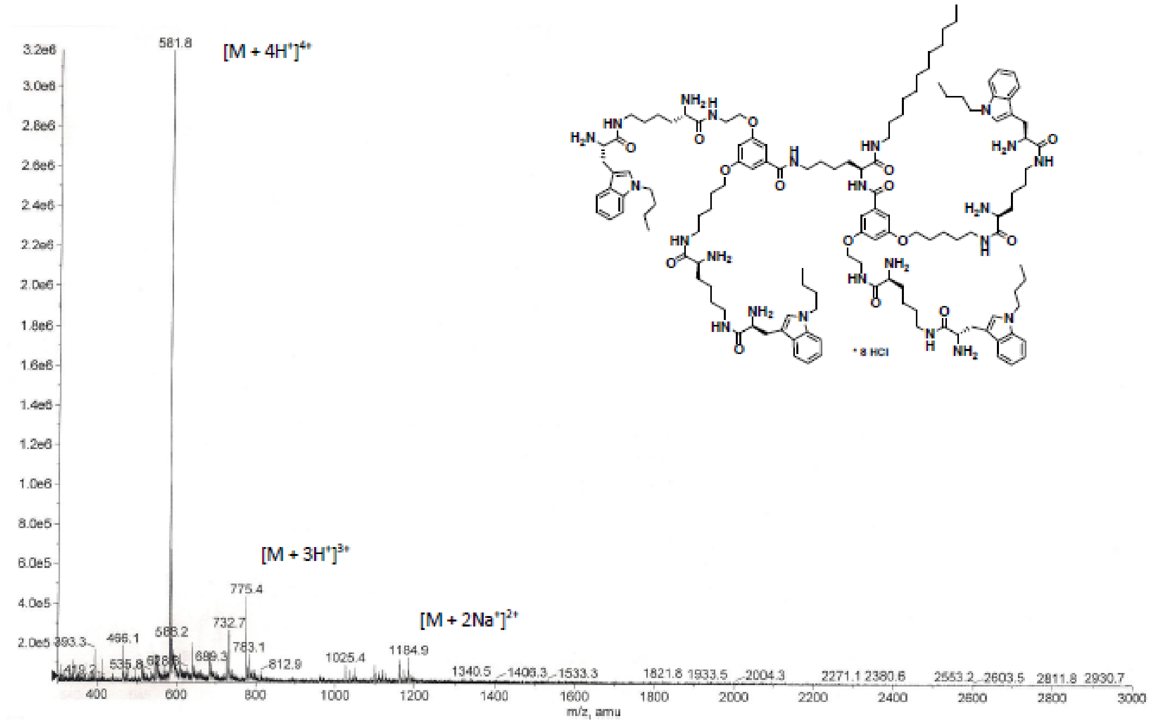

LRMS (ESI, MeOH): 1184.9 $[M + 2Na^{+}]^{2+}$, 775.4 $[M + 3H^{+}]^{3+}$, 581.7 $[M + 4H^{+}]^{4+}$ - *main signals*.

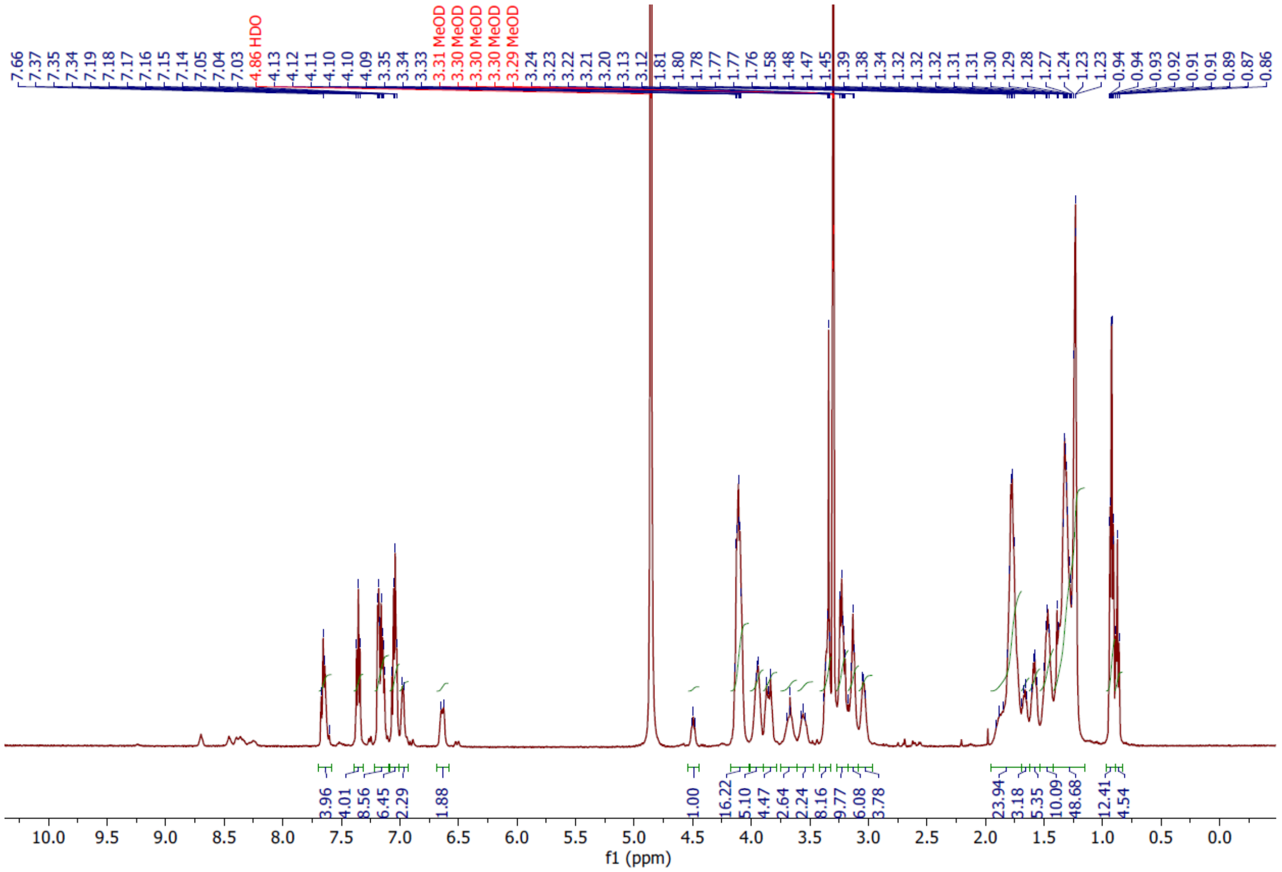

^{1}**H NMR** (500 MHz, MeOD): δ = 0.85-0.95 (2m, 15H, CH_3 *DDA*, 4×CH_3 *n-Bu*), 1.20-1.93 [br m, 78H, 5×β, γ, δCH_2 *L-Lys* and *core*, 2×O-CH_2-(C***H₂***)$_3$-CH_2-NH, 4×CH_2-2,3 *n-Bu*, CH_2-2-11 *DDA*], 3.00-3.34 [br m, 24H, 5×εCH_2 *L-Lys i core*, βCH_2 *B-Trp*, 2×O-$(CH_2)_4$-C$\boldsymbol{H_2}$-NH, CH_2-1 *DDA*], 3.56, 3.67 (2m, 4H, 2×O-CH_2-C$\boldsymbol{H_2}$-NH), 3.85 (m, 4H, 4×αCH *L-Lys*), 3.95 [m, 4H, 2×O-C$\boldsymbol{H_2}$-$(CH_2)_4$-

NH], 4.12 (m, 16H, 4×αCH *B-Trp*, 2×O-C$\boldsymbol{H_2}$-CH_2-NH, 4×CH_2-1 *n-Bu*), 4.50 (m, 1H, αCH *core*), 6.64 (m, 2H, C^4-H *Ph*), 6.98 (m, 2H, $C^{2,6}$-H *Ph*), 7.04 (m, 6H, $C^{2,6}$-H *Ph*, C^5-H *B-Trp*), 7.17 (m, 8H, $C^{2,6}$-H *B-Trp*), 7.36 (m, 4H, C^7-H *B-Trp*), 7.65 (m, 4H, C^4-H *B-Trp*).

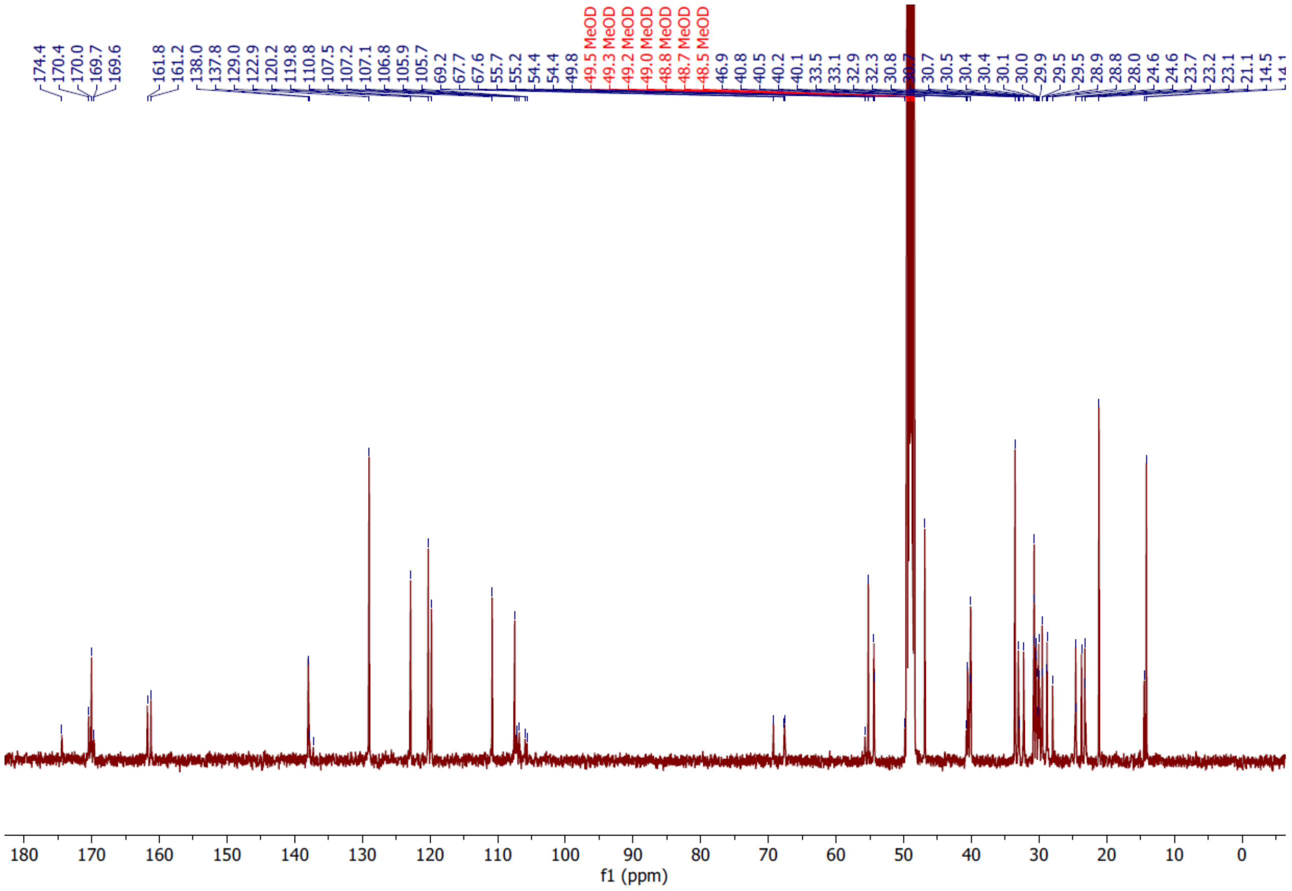

^{13}C NMR (125 MHz, MeOD): δ = 14.1 (C^4 *n-Bu*), 14.5 (C^{12} *DDA*), 21.1 (C^3 *n-Bu*), 23.1, 23.2, 23.7, 24.6 [γC, C^{11} *DDA*, 2×O-$(CH_2)_2$-$\boldsymbol{C}H_2$-$(CH_2)_2$-NH], 28.0 (C^3 *DDA*), 28.8, 28.9 (βC *B-Trp*), 29.5, 29.9, 30.1, 30.4-30.8 [δC, C^2, C^4-C^{10} *DDA*, 2×O-CH_2-$\boldsymbol{C}H_2$-CH_2-$\boldsymbol{C}H_2$-CH_2-NH], 32.3, 32.9, 33.1, 33.5 (βC, C^2 *n-Bu*), 40.1 (εC *L-Lys*), 40.2 (2×O-CH_2-$\boldsymbol{C}H_2$-NH), 40.6 [2×O-$(CH_2)_4$-$\boldsymbol{C}H_2$-NH, C^1 *DDA*], 40.8 (εC *core*), 46.9 (CH_2-1 of n-Bu), 54.4 (4×αC *L-Lys*), 55.2 (4×αC *B-Trp*), 55.7 (αC *core*), 67.6 (2×O-$\boldsymbol{C}H_2$-CH_2-NH), 69.2 [2×O-$\boldsymbol{C}H_2$-$(CH_2)_4$-NH], 105.7, 106.0 (C^4 *Ph*), 106.8, 107.2, 107.5 ($C^{2,6}$ *Ph*), 110.8 (C^7 *B-Trp*), 119.8 (C^4 *B-Trp*), 120.2 (C^5 *B-Trp*), 122.9 (C^6 *B-Trp*), 129.0 (C^2 *B-Trp*), 137.8 (C^1 *Ph*), 138.0 (C^{7a} *B-Trp*), 161.2, 161.8 ($C^{3,5}$ *Ph*), 169.6 (CONH *Ph*), 170.0, 170.5, 174.4 (CONH).

$[\alpha]_D^{25}$ = +12.3 (c 1, MeOH).

m.p.: 174-177°C.

Dendrimer 21

Dendrimer **21** was obtained from 0.42 g (0.127 mmol) of **18** in the form of a pale yellow powder ; yield 95.8% (0.34 g) z 0.42 g.

$C_{142}H_{223}O_{15}N_{23}$×8HCl, M = 2784.12 (monoisotopic mass of non-protonated dendrimer - 2492.4).

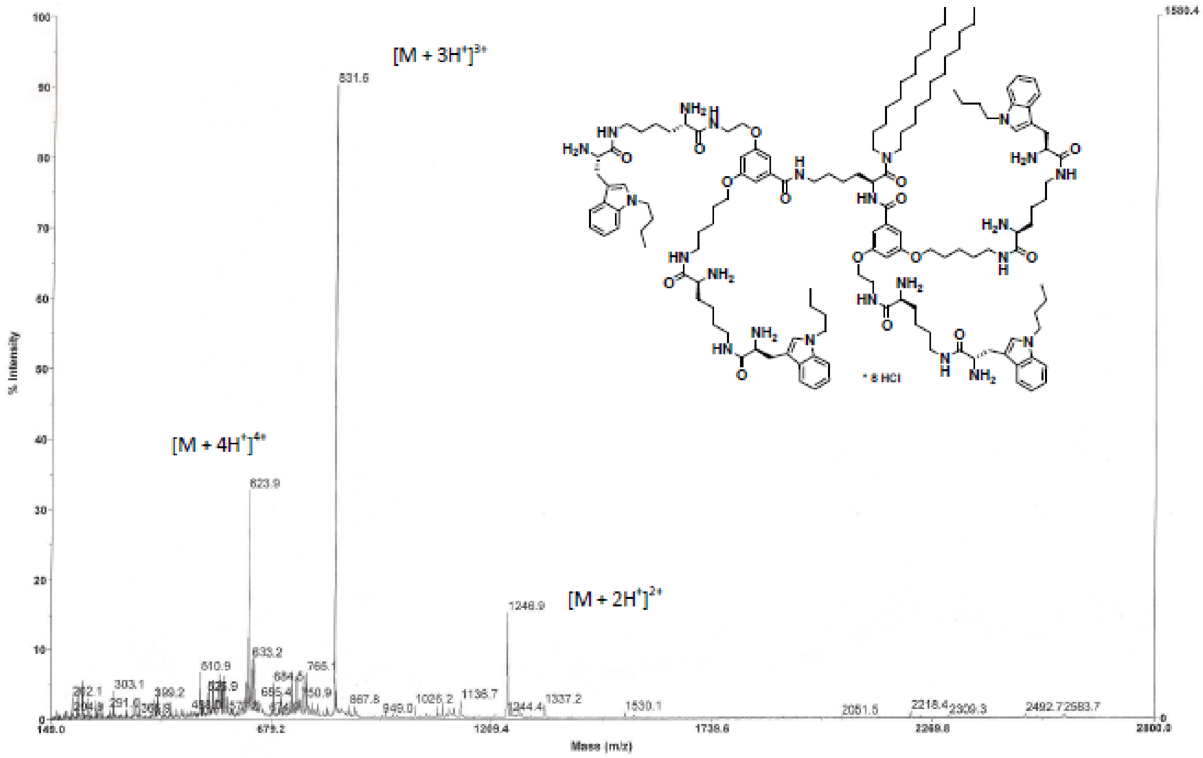

LRMS (ESI, MeOH): 1246.4 $[M + 2H^+]^{2+}$, 831.2 $[M + 3H^+]^{3+}$ - *main signals*, 623.7 $[M + 4H^+]^{4+}$.

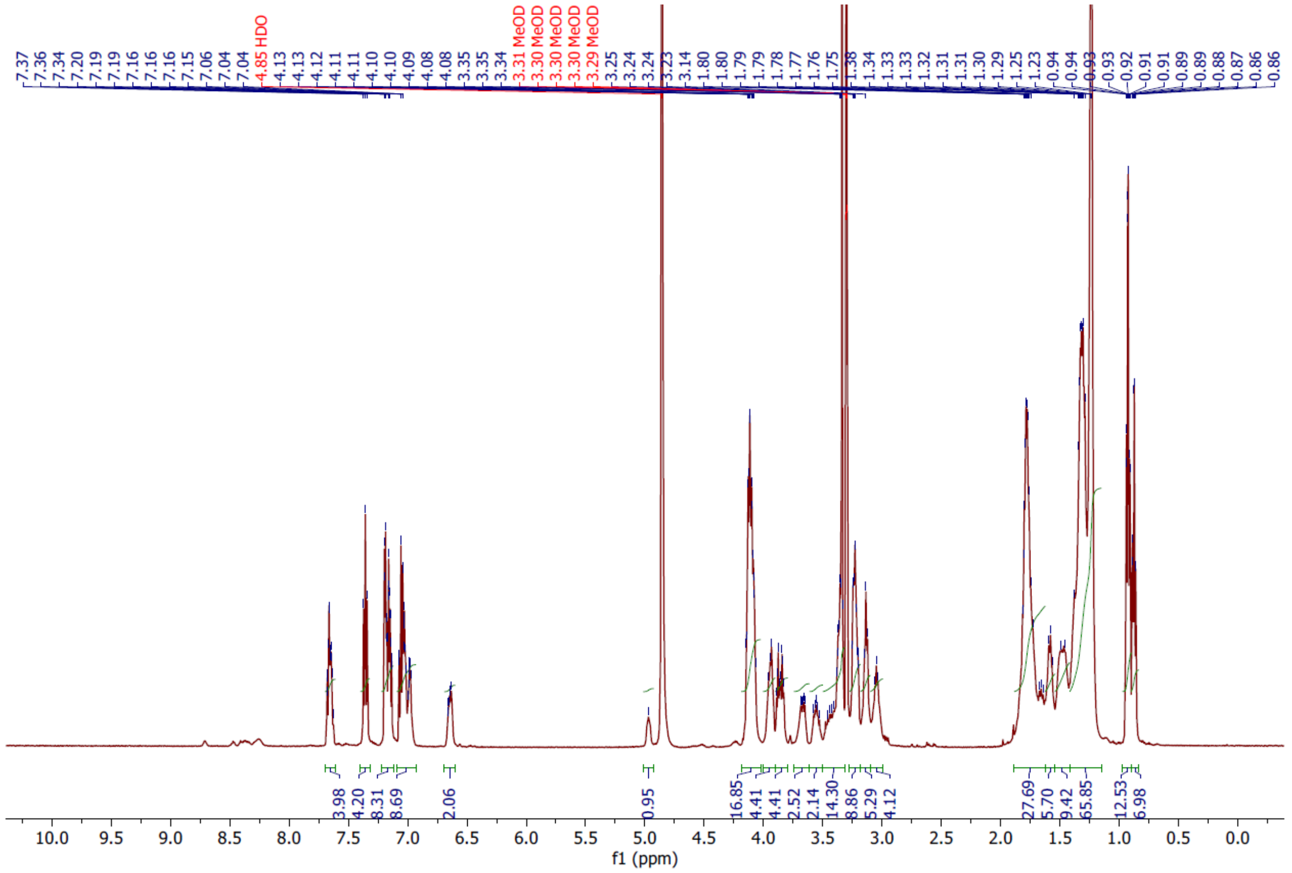

^{1}H NMR (500 MHz, MeOD): δ = 0.88 (m, 6H, 2×CH_3 *dDDA*), 0.93 (m, 12H, 4×CH_3 *n-Bu*), 1.20-1.90 [br m, 98H, 5×β, γ, δCH_2 *L-Lys* and *core*, 2×O-CH_2-(C***H$_2$***)$_3$-CH_2-NH, 4×CH_2-2,3 *n-Bu*, 2×CH_2-2-11 *dDDA*], 3.00-3.49 [br m, 26H, 5×εCH_2 *L-Lys* and *core*, βCH_2 *B-Trp*, 2×O-$(CH_2)_4$-C***H$_2$***-

NH, 2×CH_2-1 *dDDA*], 3.56, 3.67 (2m, 4H, 2×O-CH_2-C***H*****2**-NH), 3.86 (m, 4H, 4×αCH *L-Lys*), 3.94 [m, 4H, 2×O-C***H*****2**-$(CH_2)_4$-NH], 4.11 [m, 16H, 4×αCH *B-Trp*, 2×O-C***H*****2**-CH_2-NH, 4×CH_2-1 *n-Bu*], 4.97 (m, 1H, αCH *core*), 6.65 (m, 2H, C^4-H *Ph*), 6.95-7.08 (br m, 8H, $C^{2,6}$-H *Ph*, C^5-H *B-Trp*), 7.17 (m, 8H, $C^{2,6}$-H *B-Trp*), 7.36 (m, 4H, C^7-H *B-Trp*), 7.65 (m, 4H, C^4-H *B-Trp*).

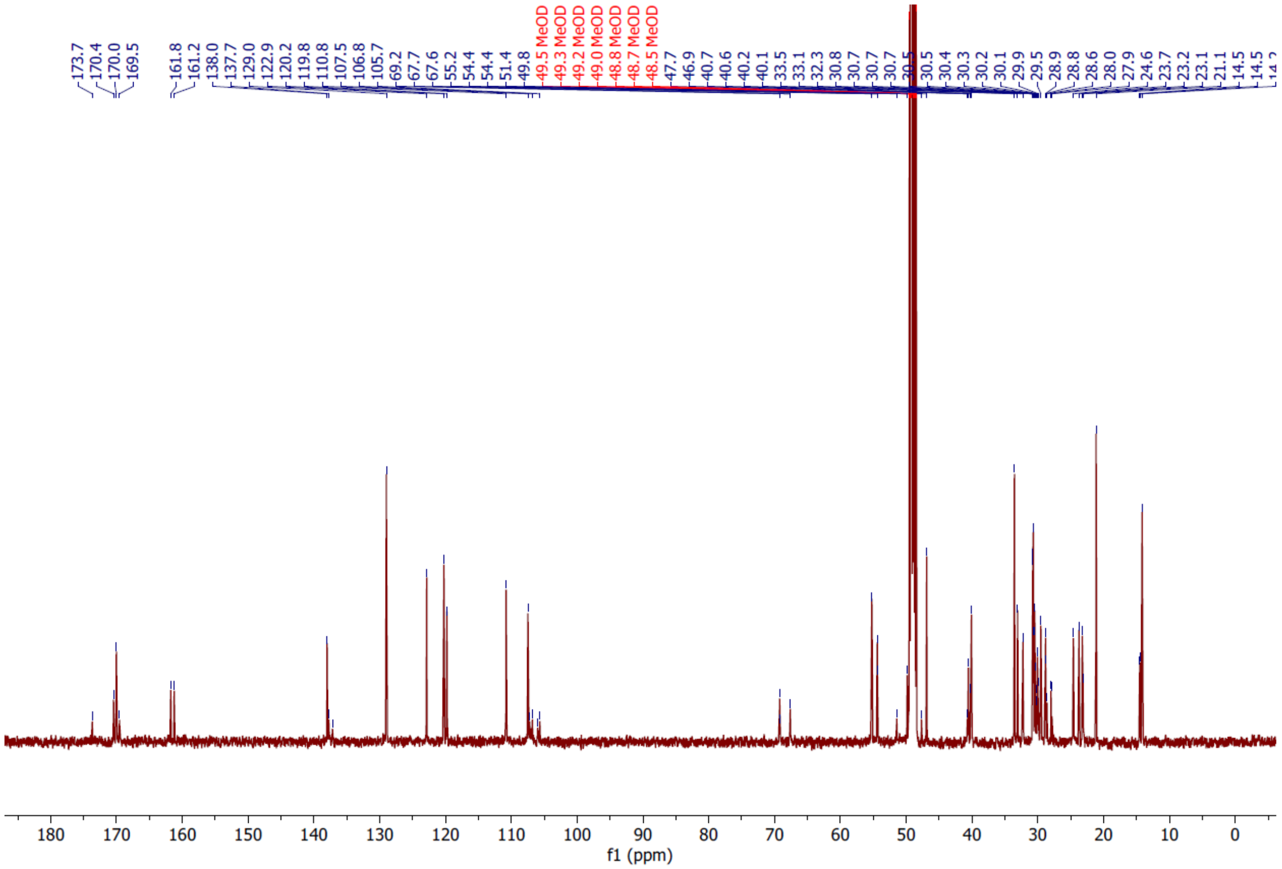

^{13}C NMR (125 MHz, MeOD): δ = 14.2 (C^4 *n-Bu*), 14.5 (2×C^{12} *dDDA*), 21.2 (C^3 *n-Bu*), 23.1, 23.2, 23.7, 23.8, 24.6 [γC, 2×C^{11} *dDDA*, 2×O-$(CH_2)_2$-***C***H_2-$(CH_2)_2$-NH], 27.9, 28.0 (2×C^3 *dDDA*), 28.6, 28.8 (βC *B-Trp*), 29.5, 30.0, 30.1, 30.2, 30.4, 30.5-30.8 [δC, 2×C^2, 2×C^4-C^{10} *dDDA*, 2×O-CH_2-***C***H_2-CH_2-***C***H_2-CH_2-NH], 32.3, 33.1, 33.6 (βC, C^2 *n-Bu*), 40.1 (εC *L-Lys*), 40.2 (2×O-CH_2-***C***H_2-NH), 40.6 [2×O-$(CH_2)_4$-***C***H_2-NH], 40.7 (εC *core*), 46.9 (C^1 *n-Bu*), 47.7 (2×C^1 *dDDA*), 51.4 (αC *core*), 54.4 (4×αC *L-Lys*), 55.2 (4×αC *B-Trp*), 67.7 (2×O-***C***H_2-CH_2-NH), 69.2 [2×O-***C***H_2-$(CH_2)_4$-NH], 105.8, 106.0 (C^4 *Ph*), 106.8, 107.2, 107.5 ($C^{2,6}$ *Ph*), 110.8 (C^7 *B-Trp*), 119.8 (C^4 *B-Trp*), 120.3 (C^5 *B-Trp*), 122.9 (C^6 *B-Trp*), 129.0 (C^2 *B-Trp*), 137.7 (C^1 *Ph*), 138.0 (C^{7a} *B-Trp*), 161.2, 161.8 ($C^{3,5}$ *Ph*), 169.5 (CONH *Ph*), 170.0, 170.5, 173.7 (CONH).

$[\alpha]_D^{25}$ = +8.9 (c 1, MeOH).

m.p.: 175-178°C.

Dendrimer 24

Dendrimer **24** was obtained with 97.8% (0.27 g) yield from 0.35 g (0.097 mmol) of dendrimer **22**.

$C_{132}H_{196}O_{15}N_{28}$×12HCl, M = 2852.68 g/mol (monoisotopic mass of non-protonated dendrimer - 2413.5). Mass without 12HCl M = 2415.15.

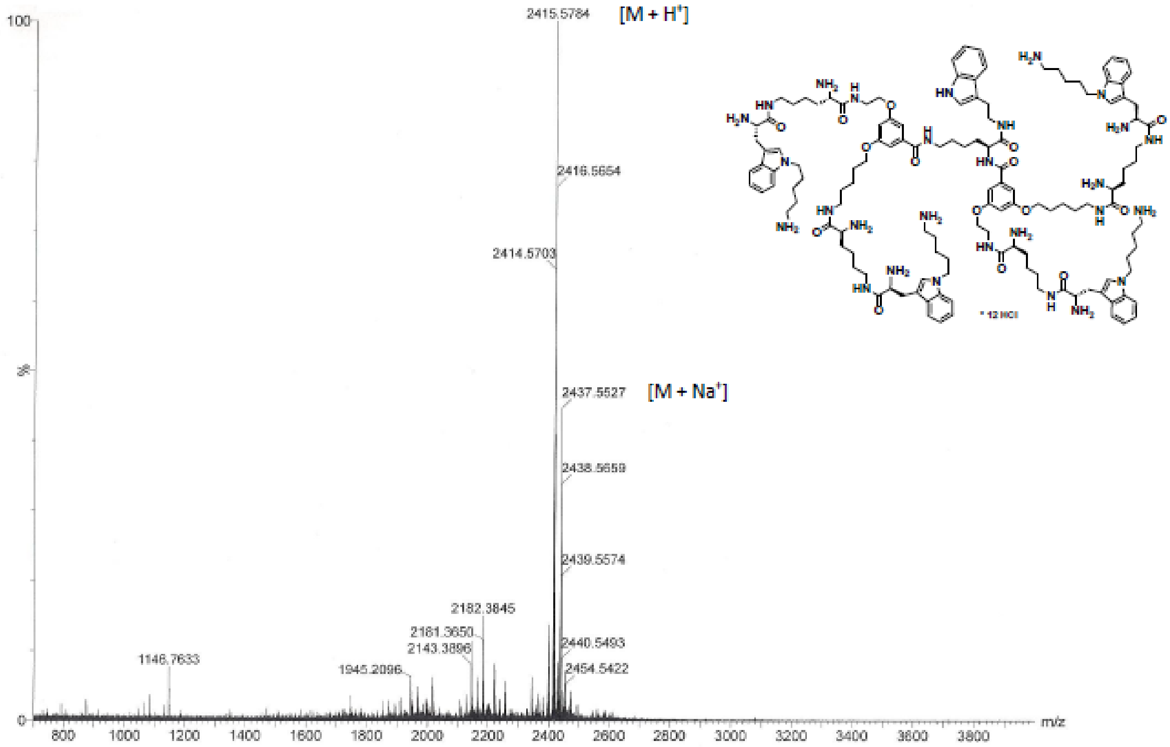

LRMS (ESI, MeOH): 617.9 $[M + MeOH + 3H^+ + Na^+]^{4+}$, 604.4 $[M + 4H^+]^{4+}$ - *main signals*; (MALDI): 2436.54 $[M + Na^+]$, 2414.57 $[M + H^+]$ - *main signals*.

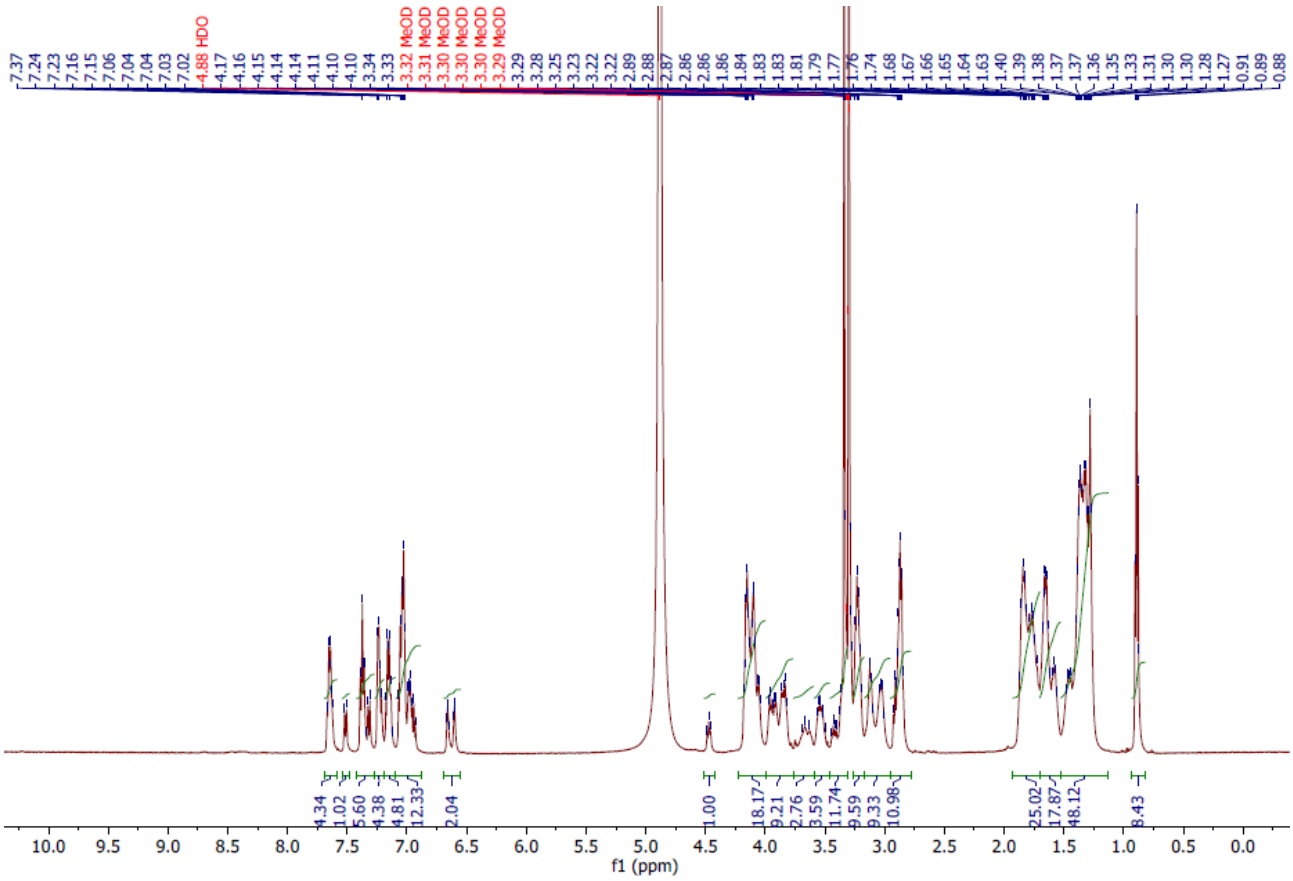

^{1}H NMR (500 MHz, MeOD): δ = 1.24-1.89 [br m, 66H, 5×β, γ, δCH_2 *L-Lys* and *core*, 2×O-CH_2-$(C\boldsymbol{H_2})_3$-CH_2-NH, 4×CH_2-2,3,4 *5-AP*], 2.87 (m, 10H, CH_2-Ar *TA*, 4×CH_2-5 *5-AP*), 2.99-3.40 [br m, 22H, 5×εCH_2 *L-Lys* and *core*, βCH_2 *AP-Trp*, 2×O-$(CH_2)_4$-$C\boldsymbol{H_2}$-NH], 3.40-3.76 (br m, 6H, 2×O-CH_2-$C\boldsymbol{H_2}$-NH, $C\boldsymbol{H_2}$-NH *TA*), 3.80-3.99 [br m, 8H, 2×O-$C\boldsymbol{H_2}$-$(CH_2)_4$-NH, 4×αCH *L-Lys*], 4.02-4.20 (br m, 16H, 2×O-$C\boldsymbol{H_2}$-CH_2-NH, 4×CH_2-1 *5-AP*, 4×αCH *AP-Trp*), 4.48 (m, 1H, αCH *core*), 6.62 (m, 2H, C^4-H *Ph*), 6.92-7.07 (br m, 11H, $C^{2,6}$-H *Ph*, C^5-H *AP-Trp*, $C^{2,5,6}$-H *TA*), 7.15 (m, 4H, C^6-H *AP-Trp*), 7.24 (m, 4H, C^2-H *AP-Trp*), 7.32 (d, J = 8.08 Hz, 1H, C^7-H *TA*), 7.37 (m, 4H, C^7-H *AP-Trp*), 7.51 (d, J = 7.84 Hz, 1H, C^4-H *TA*), 7.65 (m, 4H, C^4-H *AP-Trp*).

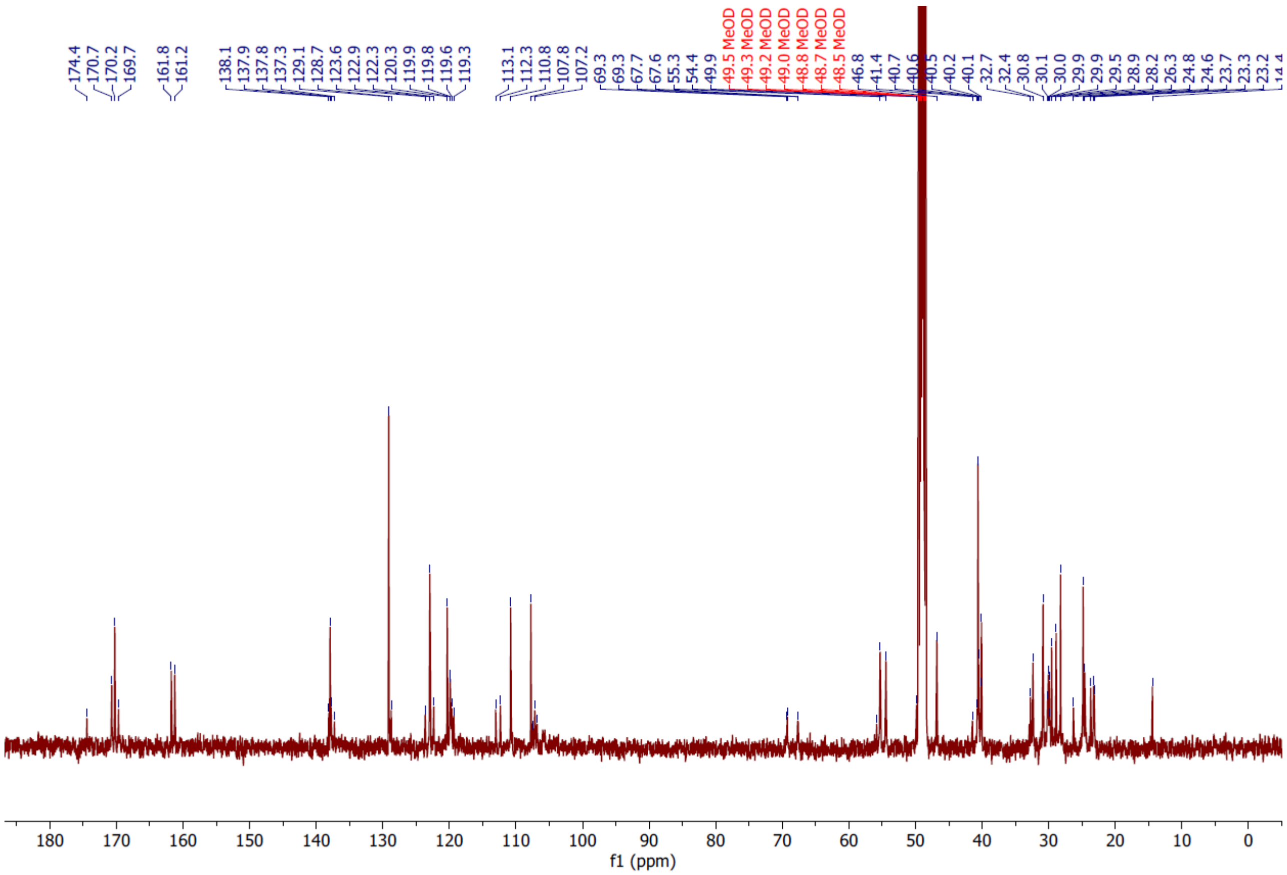

^{13}C NMR (125 MHz, MeOD): δ = 23.3, 23.7, 24.6 [γC, 2×O-$(CH_2)_2$-$\boldsymbol{C}H_2$-$(CH_2)_2$-NH], 24.8 (C^3 *5-AP*), 26.3 ($\boldsymbol{C}H_2$-Ar *TA*), 28.2 (δC), 28.9 (βC *AP-Trp*), 29.6, 29.9-30.9 [δC, $C^{2,4}$ *5-AP*, 2×O-CH_2-$\boldsymbol{C}H_2$-CH_2-$\boldsymbol{C}H_2$-CH_2-NH], 32.4, 32.8 (βC), 40.1-40.6 [2×O-CH_2-$\boldsymbol{C}H_2$-NH, 2×O-$(CH_2)_4$-$\boldsymbol{C}H_2$-NH, εC, C^5 *5-AP*], 41.5 (CH_2-NH *TA*), 46.8 (C^1 *5-AP*), 54.4 (4×αC *L-Lys*), 55.3 (4×αC *AP-Trp*), 55.8 (αC *core*), 67.6 (2×O-$\boldsymbol{C}H_2$-CH_2-NH), 69.3 [2×O-$\boldsymbol{C}H_2$-$(CH_2)_4$-NH], 105.7, 106.0 (C^4 *Ph*), 106.9, 107.2, 107.53, 107.8 ($C^{2,6}$ *Ph*), 110.8 (C^7 *AP-Trp*), 112.3 (C^7 *TA*), 113.1 (C^3 *TA*), 119.3 (C^4 *TA*), 119.6 (C^5 *TA*), 119.9 (C^4 *AP-Trp*), 120.3 (C^5 *AP-Trp*), 122.3 (C^6 *TA*), 122.9 (C^6 *AP-Trp*), 123.6 (C^2 *TA*), 128.7 (C^{3a} *AP-Trp*, C^{3a} *TA*), 129.1 (C^2 *AP-Trp*), 137.3, 137.8 (C^1 *Ph*), 137.9 (C^{7a} *AP-Trp*), 138.1 (C^{7a} *TA*), 161.2, 161.8 ($C^{3,5}$ *Ph*), 169.7 (CONH *Ph*), 170.2, 170.7, 174.4 (CONH).

$[\alpha]_D^{25}$ = +6.3 (c 1, MeOH).

m.p.: 189-192°C.

Dendrimer 25

Dendrimer **25** was obtained from 0.24 g (0.066 mmol) of dendrimer **23** in the form of pale yellow powder; yield 89.5% (0.17 g).

$C_{134}H_{211}O_{15}N_{27}$×12HCl, M = 2877.81 (monoisotopic mass of non-protonated dendrimer 2438.7). Mass without 12HCl M = 2440.28.

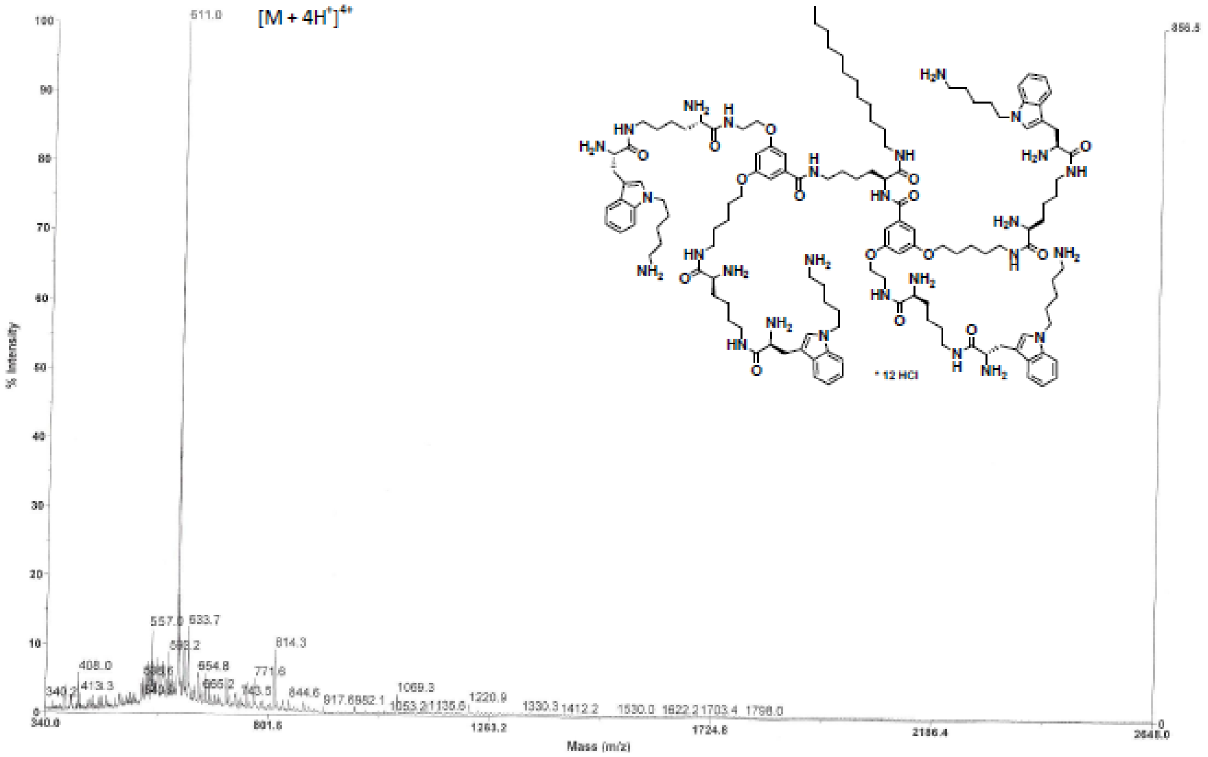

LRMS (ESI, MeOH): 610.7 $[M + 4H^{+}]^{4+}$.

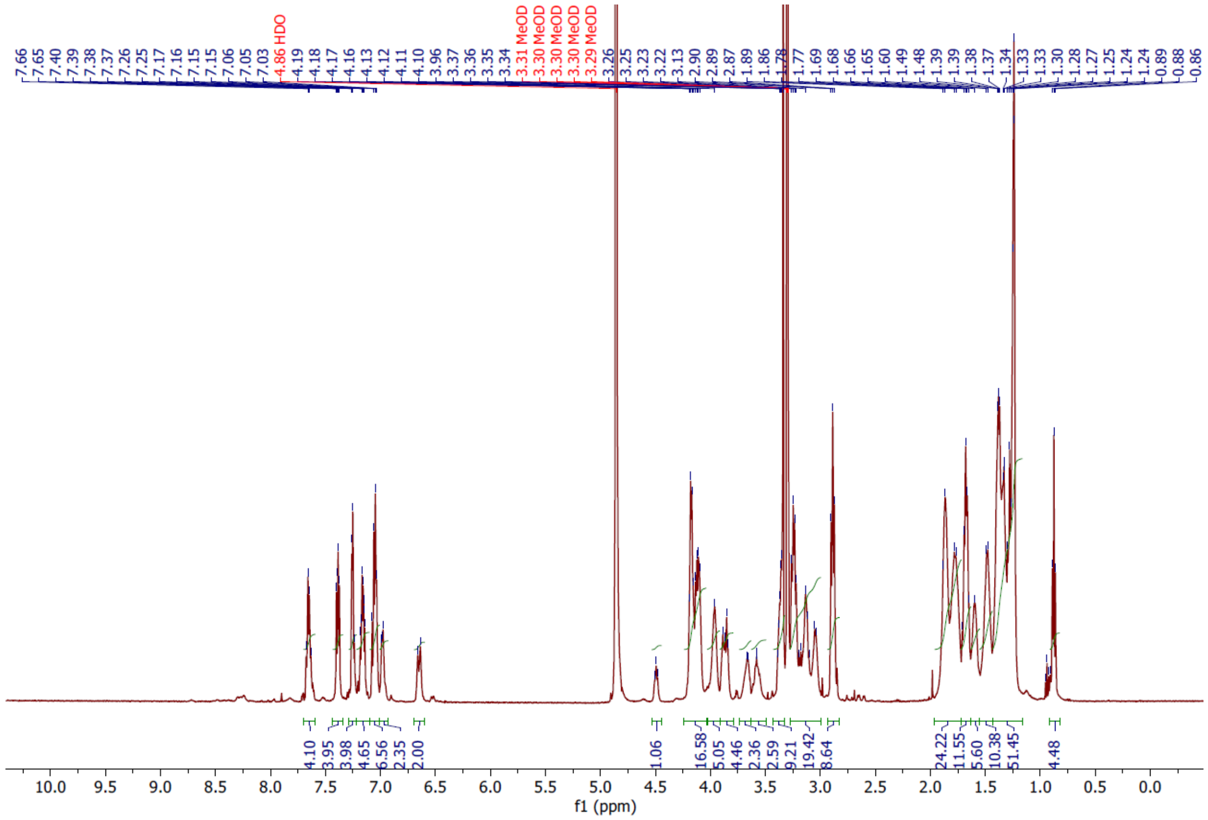

^{1}H NMR (500 MHz, MeOD): δ = 0.88 (br t, 3H, CH_3 *DDA*), 1.20-1.93 (br m, 86H, 5×γ, δ, βCH_2 *L-Lys* and *core*, CH_2-2-11 *DDA*, 2×O-CH_2-(C***H₂***)$_3$-CH_2-NH, 4×CH_2-2,3,4 *5-AP*), 2.89 (br t, 8H, 4×CH_2-5 *5-AP*), 2.90-3.40 [br m, 24H, 5×εCH_2 *L-Lys* and *core*, βCH_2 *AP-Trp*, 2×O-(CH_2)$_4$-C***H₂***-NH, CH_2-1 *DDA*], 3.58, 3.66 (2m, 4H, 2×O-CH_2-C***H₂***-NH), 3.87 (m, 4H, 4×αCH *L-Lys*), 3.97 [m, 4H, 2×O-C***H₂***-(CH_2)$_4$-NH], 4.07-4.22 (br m, 16H, 2×O-C***H₂***-CH_2-NH, 4×CH_2-1 *5-AP*, 4×αCH *AP-Trp*), 4.50 (m, 1H, αCH *core*), 6.65 (m, 2H, C^4-H *Ph*), 6.98 (m, 2H, $C^{2,6}$-H *Ph*), 7.05 (m, 6H, $C^{2,6}$-H *Ph*, C^5-H *AP-Trp*), 7.16 (m, 4H, C^6-H *AP-Trp*), 7.25 (m, 4H, C^2-H *AP-Trp*), 7.39 (m, 4H, C^7-H *AP-Trp*), 7.66 (m, 4H, C^4-H *AP-Trp*).

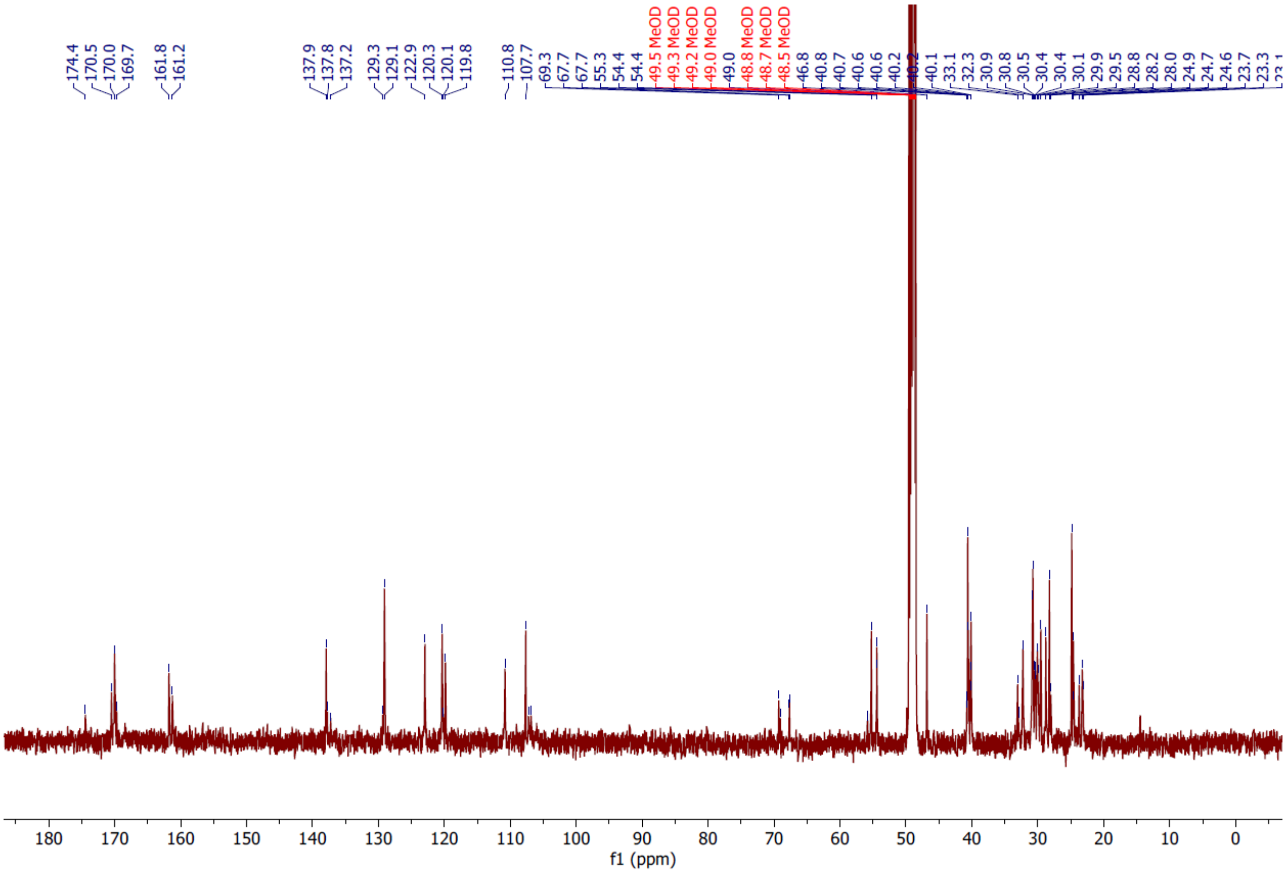

13*C NMR** (125 MHz, MeOD): δ = 14.5 (C^{12} *DDA*), 23.1, 23.3, 23.7, 24.6 [γC, 2×O-$(CH_2)_2$-C***H_2-$(CH_2)_2$-NH, C^{11} *DDA*], 24.9 (C^3 *5-AP*), 28.0 (C^3 *DDA*), 28.2 (δC), 28.8 (βC *AP-Trp*), 29.5, 30.1-30.9 [δC, $C^{2,4}$ *5-AP*, 2×O-CH_2-***C***H_2-CH_2-***C***H_2-CH_2-NH, C^2, C^4-C^{10} *DDA*], 32.3, 33.1 (βC), 40.1-40.6 [2×O-CH_2-***C***H_2-NH, 2×O-$(CH_2)_4$-***C***H_2-NH, εC, C^5 *5-AP*, C^1 *DDA*], 46.8 (C^1 *5-AP*), 54.4 (4×αC *L-Lys*), 55.3 (4×αC *AP-Trp*), 55.8 (αC *core*), 67.7 (2×O-***C***H_2-CH_2-NH), 69.3 [2×O-***C***H_2-$(CH_2)_4$-NH], 105.7, 106.0 (C^4 *Ph*), 106.8, 107.1, 107.5, 107.7 ($C^{2,6}$ *Ph*), 110.8 (C^7 *AP-Trp*), 119.8 (C^4 *AP-Trp*), 120.3 (C^5 *AP-Trp*), 122.9 (C^6 *AP-Trp*), 129.1 (C^2 *AP-Trp*), 137.9 (C^{7a} *AP-Trp*), 161.2, 161.8 ($C^{3,5}$ *Ph*), 170.0, 170.5 (CONH).

$[\alpha]_D^{25}$ = +6.6 (c 1, MeOH).

m.p.: 184-187˚C.
